# Anion–π catalysis: bicyclic products with four contiguous stereogenic centers from otherwise elusive diastereospecific domino reactions on π-acidic surfaces†
†Electronic supplementary information (ESI) available: Detailed procedures and results for all reported experiments. See DOI: 10.1039/c7sc00525c



**DOI:** 10.1039/c7sc00525c

**Published:** 2017-03-17

**Authors:** Le Liu, Yoann Cotelle, Juliane Klehr, Naomi Sakai, Thomas R. Ward, Stefan Matile

**Affiliations:** a Department of Organic Chemistry , University of Geneva , Geneva , Switzerland . Email: stefan.matile@unige.ch ; www.unige.ch/sciences/chiorg/matile/ ; Tel: +41 22 379 6523; b National Centre of Competence in Research (NCCR) Molecular Systems Engineering , Switzerland . www.nccr-mse.ch; c Department of Chemistry , University of Basel , Basel , Switzerland

## Abstract

Anion–π interactions have been introduced recently to catalysis. The idea of stabilizing anionic intermediates and transition states on π-acidic surfaces is a new fundamental concept. By now, examples exist for asymmetric enolate, enamine, iminium and transamination chemistry, and the first anion–π enzyme has been created. Delocalized over large aromatic planes, anion–π interactions appear particularly attractive to stabilize extensive long-distance charge displacements during domino processes. Moving on from the formation of cyclohexane rings with five stereogenic centers in one step on a π-acidic surface, we here focus on asymmetric anion–π catalysis of domino reactions that afford bicyclic products with quaternary stereogenic centers. Catalyst screening includes a newly synthesized, better performing anion–π version of classical organocatalysts from cinchona alkaloids, and anion–π enzymes. We find stereoselectivities that are clearly better than the best ones reported with conventional catalysts, culminating in unprecedented diastereospecificity. Moreover, we describe achiral salts as supramolecular chirality enhancers and report the first artificial enzyme that operates in neutral water with anion–π interactions, *i.e.*, interactions that are essentially new to enzymes. Evidence in support of contributions of anion–π interactions to asymmetric catalysis include increasing diastereo- and enantioselectivity with increasing rates, *i.e.*, asymmetric transition-state stabilization in the presence of π-acidic surfaces and inhibition with the anion selectivity sequence NO_3_^–^ > Br^–^ > BF_4_^–^ > PF_6_^–^.

## Abstract 2

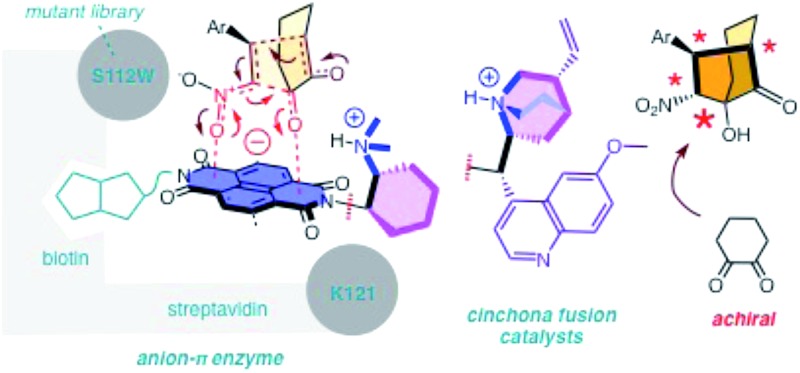
Delocalized over aromatic planes, anion–π interactions emerge as best to stabilize long-distance charge displacements in domino reactions of highest sophistication.

Contributions of anion–π interactions^1,2^ to catalysis were first demonstrated explicitly in 2013.^3^ Since then, anion–π catalysis has been explored with enolate,^4,5^ enamine,^6^ iminium,^7^ transamination^8^ and oxocarbenium^9^ chemistry, and the first anion–π enzyme has been created.^5^ The idea of stabilizing anionic intermediates and transition states on π-acidic surfaces is a new fundamental concept. Delocalized over large aromatic planes, anion–π interactions appear particularly advantageous to stabilize extensive charge displacements over long distances. For the conventional cation–π catalysis, this advantage is best illustrated with the stabilization of carbocations moving along the emerging rings during the cyclization of terpenes into steroids.^10^ In anionic tandem, domino or cascade reactions, the key intermediates in need of stabilization are not carbocations but enolates, nitronates, and so on. The most sophisticated domino reaction catalyzed so far with anion–π interactions is the stereoselective formation of a cyclohexane ring from achiral starting materials.^7^ From there, a continuing increase in sophistication of long-distance cascade charge displacements on π-acidic surfaces naturally leads to bicyclic products on the one hand and quaternary stereogenic centers on the other. For this purpose, we focused first on the addition of cyclohexanedione **1** to nitroolefin **2** (Fig. 1). In the presence of a base, they engage in a domino Michael–Henry reaction to afford bicyclo[3.2.1]octan-8-one **3**, which is a bicyclic product with four chiral centers made from achiral substrates.^11–13^ The first step is the Michael addition of the conjugate enolate base of **1** to acceptor **2** (see transition state **TS1**, Fig. 1). The resulting nitronate engages in an intramolecular Henry reaction to close the second carbocycle (**TS2**, Fig. 1). This reaction was attractive for anion–π catalysis because stabilization of the anionic enolate and nitronate intermediates on π-acidic surfaces was conceivable, and the diastereoselectivity reported in the literature for metal-free organocatalysts (up to 12 : 1) appeared improvable.^11–13^ One report highlights that poor 1 : 3 *dr* originates from epimerization between **3** and **3d** in the presence of base catalysts.^11^ Here, we report that domino catalysis on π-acidic surfaces provides access to diastereospecificity, *i.e.*, the exclusive formation of one diastereomer. This breakthrough with the most sophisticated tandem process realized so far is achieved with three different functional systems, *i.e.*, new anion–π cinchona fusion catalysts, anion–π enzymes and achiral anion–π chirality enhancers. Decisive contributions from anion–π interactions could be deduced from the observed increase in rate and stereoselectivity upon attachment of π-acidic surface, and selective inhibition with anions.

**Fig. 1:**
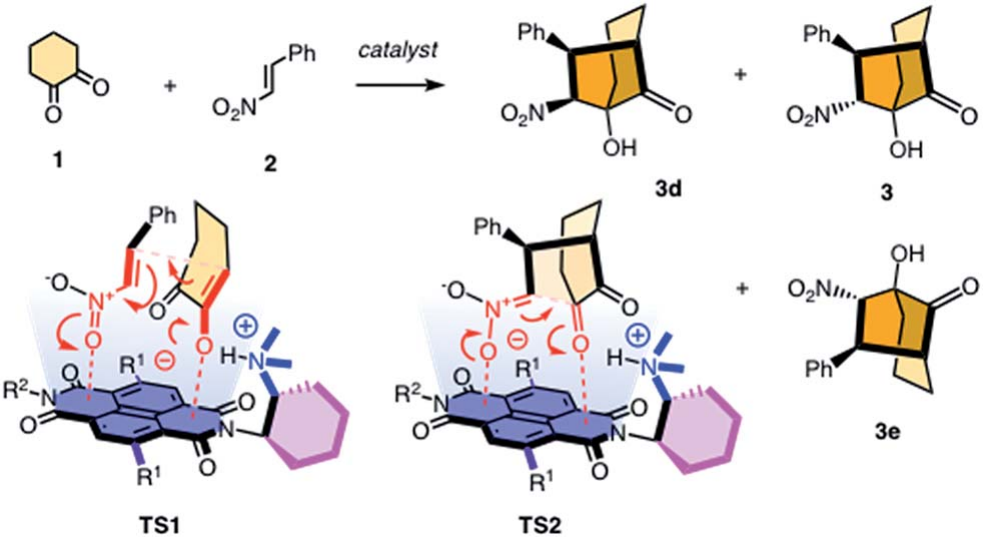
The reaction selected for anion–π catalysis of cascade reactions to bicyclic products, with notional structures for the stabilization of the anionic transition state **TS1** and **TS2** on the π-acidic surface of naphthalenediimides; for *R* ^1^ and *R* ^2^, see Fig. 2.

To elaborate on the formation of bicyclic products from achiral diketones on π-acidic surfaces, catalysts and controls **4–14** were prepared (Fig. 2). Most were accessible following reported procedures; details can be found in the ESI (Schemes S1–S5†). Anion–π catalyst **4** has been designed around the π-acidic surface of a naphthalenediimide (NDI).^2^ NDIs offer a privileged platform in anion–π catalysis because their intrinsic quadrupole moment perpendicular to the π surface is very high and can be easily modulated with substituents in the core.^2–8^ In anion–π catalyst **4**, this π surface was connected to a tertiary amine catalyst *via* a fixed Leonard turn.^4^ These turns have been introduced to assure that the reactions really occur on the π surfaces and benefit best from anion–π interactions, even when they are not particularly strong.^4^ The imide on the other side of the π-acidic surface continues with a simple solubilizing group.

**Fig. 2:**
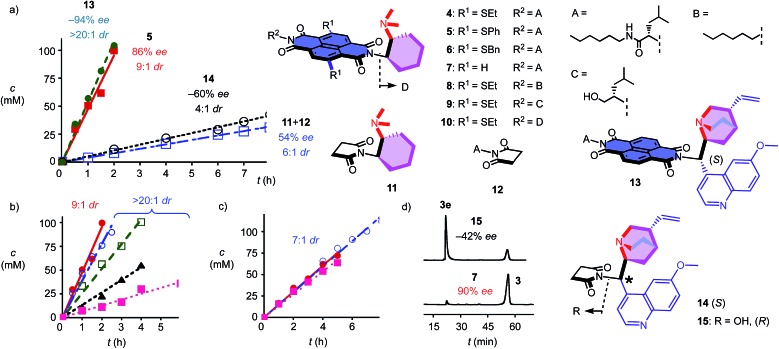
Structure of original anion–π catalysts and controls, and product concentration as a function of time (a) in the presence of catalysts **13** (), **5** (■), **14** (○) and **11** + **12** (□), (b) with **5** () and 1.2 M TBAPF_6_ (○), TBABF_4_ (□), TBABr (▲) or TBANO_3_ (■) and (c) with **11** + **12** () and 1.2 M TBAPF_6_ (■) or TBANO_3_ (○). (d) Chiral HPLC analyses for product enantiomers **3e** (first peak) and **3** (second) obtained with **15** (top) and **7** (bottom).

In the presence of 10 mol% of anion–π catalyst, the formation of bicyclo[3.2.1]octan-8-one **3** occurred within 1–3 days at ambient temperature. The known absolute configuration of product **3e** obtained with quinine **15** allowed us to assign the absolute configuration of enantiomer **3** obtained with **4–12** (Fig. 2d).^12^ Solvent screening with **4** gave best results in C_6_F_6_, *i.e.* 10 : 1 *dr* and 80% *ee* (Table 1, entries 12–15). With 20% C_6_D_6_, a slight increase to 13 : 1 *dr* coincided with a slight decrease to 77% *ee* (Table 1, entry 7). Further decreasing π acidity of the solvent gradually decreased stereoselectivity down to 6 : 1 *dr* and 66% *ee* in C_6_D_6_ (Table 1, entry 4). These trends were interesting because they supported contributions from synergistic anion–π interactions with polarization of the NDI plane induced by the solvent.^14^ From C_6_F_6_, enantioselectivities could be further increased with co-solvents, best with 88% *ee* in C_6_F_6_/CDCl_3_ 2 : 1, but diastereoselectivity dropped to 7 : 1 *dr* under these conditions (Table 1, entry 9).

**Table 1:** Catalyst performance

Entry		Cat^*a*^ (mol%)	Conditions^*b*^	*η* ^*c*^ (%)	*ee* ^*d*^ (%)	*dr* ^*e*^
1		**4** (10)	CDCl_3_	86	67	8 : 1
2		**4** (10)	THF-*d*_8_	73	79	5 : 1
3		**4** (10)	CD_3_CN	60	80	7 : 1
4		**4** (10)	C_6_D_6_	75	66	6 : 1
5		**4** (10)	Toluene-*d*_8_	83	68	10 : 1
6		**4** (10)	C_6_H_5_NO_2_	74	75	10 : 1
7		**4** (10)	C_6_F_6_/C_6_D_6_ 4 : 1	88	77	13 : 1
8		**4** (10)	C_6_F_6_/CD_3_CN 4 : 1	80	80	9 : 1
9		**4** (5)	C_6_F_6_/CDCl_3_ 2 : 1	92	88	7 : 1
10		**4** (5)	C_6_F_6_/CDCl_3_ 3 : 1	95	86	8 : 1
11		**4** (5)	C_6_F_6_/CDCl_3_ 4 : 1	91	84	9 : 1
12		**4** (10)	C_6_F_6_	93	80	10 : 1
13		**4** (5)	C_6_F_6_	92	78	10 : 1
14		**4** (2.5)	C_6_F_6_	92	78	10 : 1
15		**4** (5)	C_6_F_6_, 5 °C	95	79	13 : 1
16		**5** (5)	C_6_F_6_	91	83	13 : 1
17		**6** (5)	C_6_F_6_	86	78	7 : 1
18		**7** (5)	C_6_F_6_	95	90	7 : 1
19		**9** (5)	C_6_F_6_	86	73	11 : 1
20		**11** + **12** (5)	C_6_F_6_	86	54	7 : 1
21		**8** (5)	C_6_F_6_/CDCl_3_ 2 : 1	94	89	9 : 1
22		**10** (5)	C_6_F_6_/CDCl_3_ 2 : 1	80	82	6 : 1
23		**13** (5)	C_6_F_6_/CDCl_3_ 4 : 1	89	–94	>20 : 1
24		**14** (5)	C_6_F_6_/CDCl_3_ 4 : 1	90	–60	4 : 1
25		**15** (5)	Toluene-*d*_8_	83	–42	4 : 1
26		**5** (5)	C_6_F_6_/CDCl_3_ 4 : 1	91	86	9 : 1
27		**5** (5)	+PF_6_^–^ ^*f*^	90	90	>20 : 1
28		**5** (5)	+BF_4_^–^ ^*f*^	89	85	>20 : 1
29		**5** (5)	+Br^–^ ^*f*^	94	85	>20 : 1
30		**5** (5)	+NO_3_^–^ ^*f*^	89	78	>20 : 1
31		**13** (5)	+NO_3_^–^ ^*f*^	92	–94	>20 : 1
32		**11** + **12** (10)	C_6_F_6_/CDCl_3_ 4 : 1	95	54	7 : 1
33		**11** + **12** (10)	+PF_6_^–^ ^*f*^	94	30	10 : 1
34		**11** + **12** (10)	+NO_3_^–^ ^*f*^	92	52	8 : 1
35		**16** + WT^*g*^	Buffer pH 6.5^*h*^	53	–45	>20 : 1
36		**16** + K121A^*g*^		39	0	
37		**16** + K121R^*g*^		47	–20	>20 : 1
38		**16** + S112Y^*g*^		47	–53	>20 : 1
39		**16** + S112W^*g*^		51	–76	>20 : 1
40		**16** + S112W^*g*^	+NO_3_^–^	33	–8	>20 : 1

^*a*^Catalysts (see Fig. 2) and loading.

^*b*^ **1** (entries 1–34: 0.4 M; entries 35–40: 6.7 mM), **2** (entries 1–34: 0.8 M; entries 35–40: 16.6 mM), 2.5–10 mol% catalyst, 20 °C unless stated, 1–3 days, modifications from standard conditions are indicated at first appearance.

^*c*^Yield based on crude ^1^H NMR spectroscopy with dibromomethane as an internal standard.

^*d*^Enantiomeric excess; positive values refer to **3**, negative values to **3e**, Fig. 1.

^*e*^Diastereomeric ratio, **3** *vs.* **3d** or **3e** *vs.* **3ed** (Fig. 1).

^*f*^1.2 M TBA salts.

^*g*^ **16** bound to WT or mutant streptavidin as a catalyst (5 mol%).

^*h*^33 mM Bis–Tris, 33% MeCN.

In C_6_F_6_, catalyst loadings could be reduced to 2.5 mol% without significant losses in stereoselectivity (Table 1, entries 12–14). At 5 mol% in C_6_F_6_, reduction of the temperature to 5 °C further increased diastereoselectivity to 13 : 1 *dr*, whereas enantioselectivity did not change significantly (Table 1, entry 15).

Variation of the core substituents did not affect the activity of catalysts **4–7** significantly, also because the π acidity of the π surface was not much changed (Table 1, entries 13 and 16–18).^15^ The best activity was obtained for **5** with phenylsulfides in the core, *i.e.*, 83% *ee* and 13 : 1 *dr* for 5 mol% in C_6_F_6_ (Table 1, entry 16). The 90% *ee* obtained at maximal π acidity without core substituents in **7** was accompanied by a decrease in diastereoselectivity to 7 : 1 *dr* (Table 1, entry 18). Similarly insignificant were variations of the second imide substituent, *i.e. R* ^2^ opposite to the Leonard-turned amine in catalysts **8–10** (Table 1, entries 19, 21 and 22). This trend suggested that these anion–π catalysts are formally bifunctional, but not trifunctional (Fig. 1).

Cinchona alkaloids are most popular in amine-based asymmetric organocatalysis.^11,12,16^ In the newly designed catalyst **13**, this bicyclic amine was attached to a π-acidic surface *via* a Leonard turn, similar to the one introduced in catalysts **4–10** (Fig. 2). The synthesis of the new anion–π cinchona fusion catalyst **13** was very straightforward (Scheme S4†). In C_6_F_6_/CDCl_3_ 4 : 1, 5 mol% of cinchona catalyst **13** produced bicycle **3e** in 89% yield with –94% *ee* and only one detectable diastereomer, *i.e.*, >20 : 1 *dr* (Table 1, entry 23). This shift from diastereoselectivity to diastereospecificity is unprecedented for this domino process; previous records in the literature, achieved with cinchona catalysts interfaced with conventional thioureas, stopped at 12 : 1 *dr*.^12^ The diastereospecificity obtained with cinchona catalyst **13** suggested that on π-acidic surfaces, either the epimerization between **3e** and **3ed** is suppressed or the protonation of the nitronate intermediate is stereospecific.

Cinchona controls **14** and **15** without a π-acidic surface gave only –60% *ee* (4 : 1 *dr*) and –42% *ee* (lit. –17% *ee*,^12^ Table 1, entries 24 and 25), respectively. The increase in stereoselectivity upon interfacing of cinchona alkaloids with a π-acidic surface in cinchona catalyst **13** coincided with a rate enhancement of *v*/*v*_0_ = 10 (Fig. 2a). This acceleration corresponded to a transition-state stabilization (or ground-state destabilization) of Δ*E*_a_ = –5.7 kJ mol^–1^. For comparison with Leonard catalyst **5**, controls **11** and **12** without π-acidic surface also gave much poorer stereoselectivity (54% *ee*, 7 : 1 *dr*) at a slower rate (Fig. 2a, Table 1, entries 16 and 20). The rate enhancement *v*/*v*_0_ = 12 corresponded to Δ*E*_a_ = –6.1 kJ mol^–1^. The similar transition-state stabilization obtained with the conventional Leonard catalyst **5** and new fusion catalyst **13** was meaningful considering the similarity of the π acidity of the respective π surfaces. Rate enhancements coinciding with increased stereoselectivity in the presence of π-acidic surfaces provided strong support for contributions of anion–π interactions to asymmetric catalysis.^3–8^

Corroborative support for this important conclusion was obtained by inhibition with inorganic anions. Consistent with competitive anion–π interactions, the velocity of Leonard catalyst **5** decreased in the presence of various tetrabutylammonium (TBA) salts with a selectivity sequence PF_6_^–^ < BF_4_^–^ < Br^–^ < NO_3_^–^ (Fig. 2b; Table 1, entries 27–30). A half maximal inhibitory concentration (IC_50_) of nitrate was calculated to be 0.70 M for catalyst **5** (Fig. S17†). For cinchona catalyst **13**, a similar IC_50_ = 0.75 M was obtained (Fig. S18†; Table 1, entry 31). Controls **11** and **12** were insensitive to the presence of anions (Fig. 2c, Table 1, entries 32–34). Interestingly, the diastereoselective Leonard catalyst **5** became diastereospecific in the presence of various TBA salts (Table 1, entries 26–30). The independence of this trend on the anion involved suggested that the TBA cations are responsible. A plausible explanation for this finding was that TBA cations induce the polarization of the π-acidic NDI core, comparable to the expected function of C_6_F_6_.^14^ Indeed, the observed acceleration of reactions with low concentrations of TBANO_3_ salts was consistent with this explanation (Fig. S16†). With cinchona catalyst **13** already producing bicycle **3e** diastereospecifically, the discovery of achiral salts as unprecedented supramolecular anion–π chirality enhancers for Leonard catalyst **5** suggested that both enantiomers **3** and **3e** could be obtained diastereospecifically on π-acidic surfaces.

To position anion–π catalysts within the chiral space of proteins, anion–π catalyst **4** has been coupled with a biotin.^5^ Binding of the resulting conjugate **16** to streptavidin then afforded an artificial anion–π enzyme, which operates with an essentially unknown interaction to biological enzymes (Fig. 3a).^5^ Interfacing of anion–π catalysts with proteins is attractive because access to mutant screening allows performance to be readily optimized. This screening approach has previously afforded anion–π enzymes that catalyze, at pH 3.0, the addition of malonic acid half thioesters to enolate acceptors with 95% *ee* and unprecedented chemospecificity with regard to the intrinsically favored decarboxylation.^5^ A focused mutant screening for the domino reaction to bicycle **3** in the presence of **16** gave best results for S112W at pH 6.5 (–76% *ee*, Table 1, entry 39). Mutant S112Y, the best for the addition of malonic acid half thioesters, was with –53% *ee* slightly better than the wild-type (WT) protein with –45% *ee* (Table 1, entries 35 and 38). K121 was confirmed as essential, presumably to keep the tertiary amine base in **16** from protonation under experimental conditions (Table 1, entries 36 and 37). In the presence of nitrate, the enantioselectivity decreased, with IC_50_ = 0.34 M (Fig. 3b, Table 1, entry 40). This finding provided experimental support for operational anion–π interactions, *i.e.* the existence of anion–π enzymes. Interestingly, all anion–π enzymes obtained with **16** gave enantiomer **3e** with a maximum *ee* of –76% as the main product in nearly neutral water/MeCN 2 : 1, whereas the protein-free analog **4** gave the opposite enantiomer **3** with a maximum *ee* of 88% as the main product in C_6_F_6_/CDCl_3_ 4 : 1. It is perhaps this inversion of the intrinsic enantioselectivity induced by the fixed Leonard turn derived from 1*R*,2*R*-diaminocyclohexane in both **4** and **16** that hindered access to higher *ee*’s with anion–π enzymes.

**Fig. 3:**
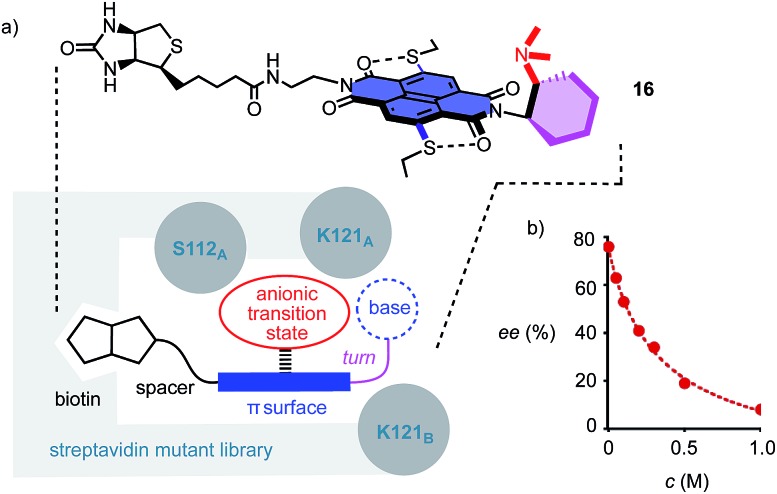
a) The concept of anion–π enzymes, with indication of the structure of the anion–π biotin conjugate **16** interface used with streptavidin mutants and the position of the mutated key residues from monomers A and B of the streptavidin tetramer, and (b) the dependence of the *ee* of **3e** produced by the S112W mutant on the concentration of NO_3_^–^ (NaNO_3_).

In summary, this study drives the development of anion–π catalysts to unprecedented sophistication with regard to anionic domino reactions that take place on π-acidic surfaces. The most important findings are anion–π cinchona fusion catalysts that clearly exceed the performance of conventional metal-free organocatalysts for the first time, the first example for diastereospecificity, anion–π enzymes that operate in neutral water and the discovery of achiral tetraalkylammonium salts as supramolecular chirality enhancers. The discovery of the best anion–π catalysts with the most sophisticated domino reaction supports the important expectation that anion–π interactions, delocalized over large aromatic planes, will be most advantageous in stabilizing extensive long-distance charge displacements during multiple coupled anionic intermediates and transition states. Long-term perspectives include the discovery of otherwise inaccessible reactions with anion–π catalysis, the integration into more complex systems,^5,17^ and the introduction of other unorthodox interactions to catalysis.^3,18^
