# Supplementary material for: Anion–π catalysis: bicyclic products with four contiguous stereogenic centers from otherwise elusive diastereospecific domino reactions on π-acidic surfaces
Source: Chem Sci. 2017 Mar 17;8(5):3770–4. doi: 10.1039/c7sc00525c (PMC5436548; doi:10.1039/c7sc00525c)
Supplement: Supplementary file 1 [file SC-008-C7SC00525C-s001.pdf]

Electronic Supplementary Material (ESI) for Chemical Science.
This journal is © The Royal Society of Chemistry 2017

Anion-π catalysis: Bicyclic products with four contiguous stereogenic centers from otherwise elusive diastereospecific domino reactions on π-acidic surfaces

Le Liu,[a] Yoann Cotelle,[a,b] Juliane Klehr,[b,c] Naomi Sakai,[a] Thomas R. Ward[b,c] and Stefan Matile*[a,b]

[a]*Department of Organic Chemistry, University of Geneva, Geneva, Switzerland.*

[b]*National Centre of Competence in Research (NCCR) Molecular Systems Engineering*

[c]*Department of Chemistry, University of Basel, Basel, Switzerland*

stefan.matile@unige.ch

Supplementary Information

Table of Content

1. Material and methods S2
2. Catalyst synthesis S3
3. Catalyst evaluation S12
4. Supplementary figures and tables S15
5. References S36
6. NMR spectra S37

1. Materials and methods

As in references S1 and S2, reagents for synthesis were purchased from Sigma-Aldrich, Fluka, Acros, Apollo Scientific and Bachem. All reactions were performed under N_2 or Ar atmosphere. Unless stated otherwise, column chromatography was carried out on silica gel 60 (SiliaFlash P60, 40-63 μm). Analytical (TLC) and preparative thin layer chromatography (PTLC) were performed on silica gel 60 (Merck, 0.2 mm) and silica gel GF (SiliCycle, 1 mm), respectively. Chiral HPLC were performed on a LC-4000 from JASCO. Melting points (Mp) were measured on a Melting Point M-565 (BUCHI). Circular dichroism spectra were obtained using JASCO J-815 spectropolarimeter and are reported as extremum wavelength λ in nm ($\Delta\varepsilon$ in $M^{-1}cm^{-1}$). UV-Vis spectra were recorded on a JASCO V-650 spectrophotometer equipped with a stirrer and a temperature controller (25 °C) and are reported as maximal absorption wavelength λ in nm (extinction coefficient ε in $M^{-1}.cm^{-1}$). IR spectra were recorded on a Perkin Elmer Spectrum One FT-IR spectrometer (ATR, Golden Gate, unless stated) and are reported as wave numbers ν in cm^{-1} with band intensities indicated as s (strong), m (medium), w (weak). ^{1}H and ^{13}C spectra were recorded (as indicated) either on a Bruker 300 MHz, 400 MHz or 500 MHz spectrometer and are reported as chemical shifts (δ) in ppm referenced to the residual solvent. Spin multiplicities are reported as a singlet (s), doublet (d), triplet (t) and quartet (q) with coupling constants (J) given in Hz, or multiplet (m). Broad peaks are marked as br. ^{1}H and ^{13}C resonances were assigned with the aid of additional information from 1D and 2D NMR spectra (H, H-COSY, DEPT 135, HSQC and HMBC). ESI-MS were performed on a ESI API 150EX and are reported as *m/z* (%). Accurate mass determinations using ESI (HR ESI-MS) were performed on a Sciex QSTAR Pulsar mass spectrometer.

Abbreviations. NDI: Naphthalenediimide; rt: Room temperature; TBA salts: Tetrabutylammonium salts; TEA: triethyl amine.

2. Catalyst synthesis

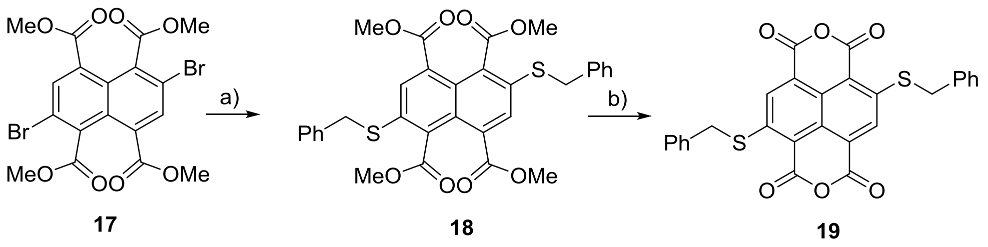

Scheme S1. Reagents and conditions: a) Cs_2CO_3, benzyl mercaptan, 1,4-dioxane/$CHCl_3$, 100 ^oC, 24 h, 53%; b) i) KOH, *i*-PrOH/H_2O, 100 ^oC, 12 h; ii) AcOH, 100 ^oC, 12 h, 65% over two steps.

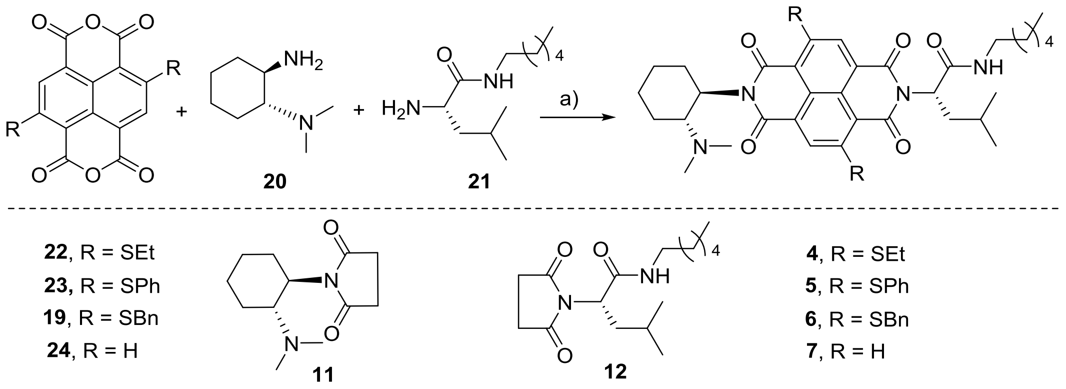

Scheme S2. Reagents and conditions: a) TEA, 1,4-dioxane, 100 ^oC, 15 h, **4**: 30%, **5**: 29%, **6**: 25%, **7**: 33%.

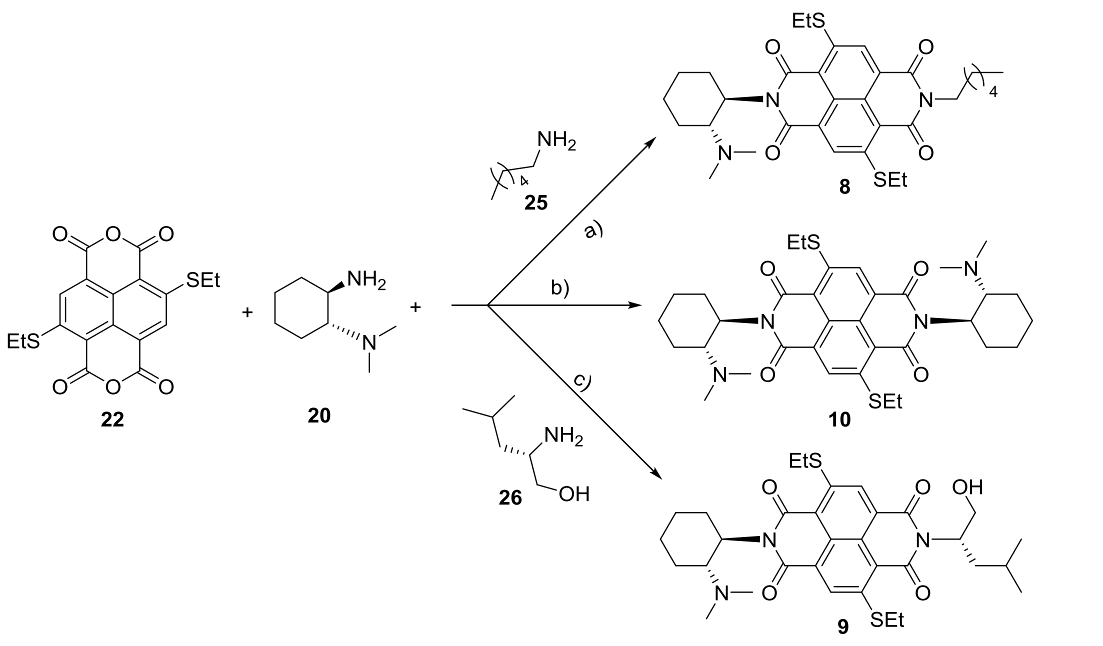

Scheme S3. Reagents and conditions: a) TEA, 1,4-dioxane, 100 ^{o}C, 15 h, 41%; b) TEA, 1,4-dioxane, 100 ^{o}C, 15 h, 70%; c) TEA, 1,4-dioxane, 100 ^{o}C, 15 h, 30%.

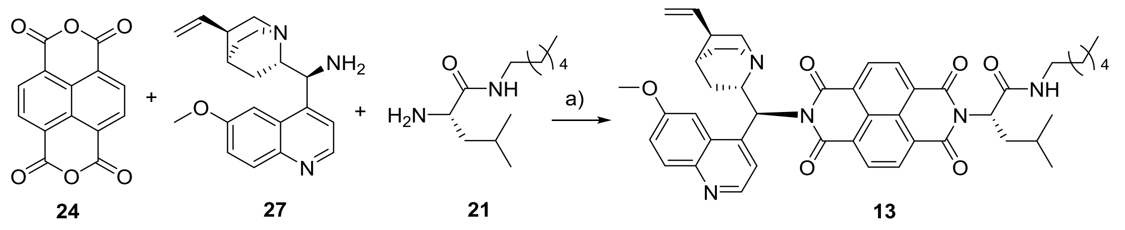

Scheme S4. Reagents and conditions: a) TEA, 1,4-dioxane, 100 ^{o}C, 12 h, 35%.

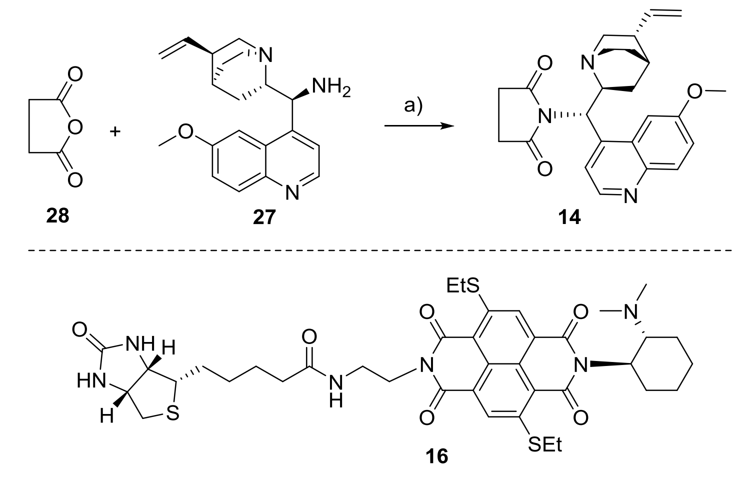

Scheme S5. Reagents and conditions: a) molecular sieves, toluene, reflux, 24 h, 70%.

Compound 4. This compound was prepared following the literature procedure.[S1]

Compound 10. This compound was prepared following the literature procedure.[S2]

Compound 11. This compound was prepared following the literature procedure.[S1]

Compound 12. This compound was prepared following the literature procedure.[S2]

Compound 16. This compound was prepared following the literature procedure.[S3]

Compound 17. This compound was prepared following the literature procedure.[S4]

Compound 20. This compound was prepared following the literature procedure.[S5]

Compound 21. This compound was prepared following the literature procedure.[S6]

Compound 22. This compound was prepared following the literature procedure.[S7]

Compound 23. This compound was prepared following the literature procedure.[S8]

Compound 27. This compound was prepared following the literature procedure.[S9]

Note: the NMR spectra of NDI compounds with cyclohexyl diamines are complex due to the presence of inseparable, inseparable rotamers.[S8] For simplification, ^{13}C NMR data presented here are only of the major peaks. The purities of the catalysts were assessed by the HPLC analyses (Figure S20).

Compound 18. To a solution of **17** (0.96 g, 1.86 mmol) in $CHCl_3$ (3 mL) and 1,4-dioxane (17 mL), Cs_2CO_3 (3.0 g, 9.3 mmol) and benzyl mercaptan (3.4 mL, 29.0 mmol) were added at rt. The resulting mixture was then heated at 100 ^oC with stirring for 24 h in a pressure-tight vessel. Upon complete consumption of the starting material indicated by TLC (R_f (pentane/EtOAc 8:2) product **18**: 0.20; substrate **17**: 0.30), the reaction mixture was allowed to cool to rt and poured into H_2O (50 mL). The mixture was extracted with CH_2Cl_2 (3 x 25 mL). The combined organic phase was washed with brine (80 mL) and dried over anhydrous Na_2SO_4. The solvent was removed under reduced pressure and the residue was purified by silica gel column

chromatography (pentane/EtOAc 9:1 then to 8:2, R_f (pentane/EtOAc 8:2): 0.20) to give **18** as a yellow solid (596 mg, 53%). Mp: 210 – 211 ºC; IR (neat): 2954 (w), 2861 (w), 1726 (s), 1704 (w), 1653 (m), 1603 (w), 1552 (w), 1438 (s), 1381 (m), 1318 (m), 1281 (m), 1242 (w), 1201 (s), 1157 (s), 1134 (s), 970 (m), 905 (w), 835 (w), 767 (m), 699 (s), 677 (m); ^{1}H NMR (300 MHz, $CDCl_3$): 7.91 (s, 2H), 7.41 – 7.34 (m, 4H),7.33 – 7.27 (m, 4H), 7.26 – 7.22 (m, 2H), 4.24 (s, 4H), 3.87 (s, 12H); ^{13}C NMR (100 MHz, $CDCl_3$): 167.7 (C), 167.1 (C), 137.7 (C), 135.8 (C), 131.4 (C), 131.1 (C), 129.9 (CH), 129.1 (CH), 128.7 (CH), 127.6 (CH), 127.2 (C), 52.5 (CH_3), 52.0 (CH_3), 38.9 (CH_2); MS (ESI, $CHCl_3$/MeOH (1:1) with 0.1% HCOOH): 573 (100, $[M\text{-}OMe]^+$), 506 (50, $[M\text{-}Bn]^+$).

Compound 19. To a solution of **18** (0.27 g, 0.45 mmol) in *i*-PrOH (20 mL) and water (20 mL), potassium hydroxide (0.76 g, 13.55 mmol) was added. The resulting mixture was heated at 100 ^{o}C for 12 h. Then, the mixture was cooled to rt and the solvent was removed under reduced pressure. The residue was dissolved in glacial acetic acid (20 mL) and heated at 100 ^{o}C for 12 h. Then, the mixture was cooled to rt and the precipitate was filtered, washed with acetic acid (20 mL) and water (30 mL), dried in vacuum to give **19** as a red solid (150 mg, 65%). It was used for the next step without further purification. IR (neat): 3029 (w), 2953 (w), 1766 (s), 1727 (s), 1600 (w), 1552 (s), 1494 (w), 1436 (m), 1421 (m), 1375 (m), 1336 (w), 1287 (m), 1238 (m), 1167 (s), 1150 (s), 1062 (s), 983 (s), 951 (w), 896 (w), 813 (w), 775 (m), 712 (m), 697 (s), 643 (m).

Compound 5 (general procedure A). To a suspension of dianhydride **23** (200 mg, 0.413 mmol) in 1,4-dioxane (10 mL) were added **20** (59 mg, 0.42 mmol) and **21** (97 mg, 0.42 mmol) at rt with stirring. Then, TEA (109 μL, 0.823 mmol) was introduced and the resulting mixture was heated at 100 ^{o}C under argon atmosphere for about 15 h. Then, the mixture was cooled to rt and concentrated *in vacuo.* Silica gel column chromatography of the residue (CH_2Cl_2/MeOH 97:3, R_f (CH_2Cl_2/MeOH 90:10): 0.50)

gave pure **5** (96 mg, 29%) as a red solid. Mp: decomp. 144 – 145 ºC; CD ($CHCl_3$): 382 (-3.82), 360 (-4.41), 329 (+3.25), 289 (+3.87); IR (neat): 3319 (w), 3067 (w), 2929 (m), 2861 (w), 2779 (w), 1696 (m), 1649 (s), 1547 (m), 1436 (s), 1370 (w), 1368 (w), 1310 (s), 1234 (s), 1214 (s), 1022 (w), 905 (w), 789 (w), 750 (m), 691 (w); ^{1}H NMR (400 MHz, $CDCl_3$): 8.13 (s, 1H), 8.10 (s, 1H), 7.65 (d, *J* = 5.6 Hz, 4H), 7.60 – 7.53 (m, 6H), 5.75 (s, 1H), 5.71 – 5.63 (m, 1H), 5.10 – 4.85 (m, 1H), 3.80 – 3.51 (m, 1H), 3.36 – 3.16 (m, 2H), 2.55 – 2.20 (m, 1H), 2.11 (s, 6H), 2.06 – 1.90 (m, 2H), 1.87 – 1.76 (m, 3H), 1.55 – 1.40 (m, 4H), 1.35 – 1.17 (m, 2H), 0.96 (d, *J* = 6.5 Hz, 3H), 0.92 (d, *J* = 6.6 Hz, 3H), 0.87 – 0.82 (m, 3H); ^{13}C NMR (100 MHz, $CDCl_3$): 169.0 (C), 163.9 (C), 163.5 (C), 162.9 (C), 162.2 (C), 149.8 (C), 149.0 (C), 148.5 (C), 136.0 (CH), 135.9 (CH), 135.8 (CH), 135.7 (CH), 130.7 (CH), 130.6 (CH), 130.4 (CH), 130.2 (CH), 129.9 (CH), 129.5 (CH), 125.4 (C), 124.3 (C), 123.4 (C), 119.9 (C), 118.0 (C), 61.7 (CH), 56.2 (CH), 53.9 (CH), 40.5 (2 x CH_3), 40.0 (CH_2), 37.7 (CH_2), 31.5 (CH_2), 29.7 (CH_2), 29.4 (CH_2), 26.5 (CH_2), 26.2 (CH_2), 25.9 (CH), 25.3 (CH_2), 23.2 (CH_3), 22.5 (CH_2), 22.2 (CH_3), 14.0 (CH_3); MS (ESI, $CHCl_3$/MeOH (1:1) with 0.1% HCOOH): 806 (100, $[M+H]^+$); HRMS (ESI, +ve) calcd for $C_{36}H_{52}N_4O_5S_2$ ($[M+H]^+$): 805.3452, found: 805.3440.

Compound 6. Following the general procedure **A**, using dianhydride **19** (170 mg, 0.332 mmol) instead of **23**, pure **6** (69 mg, 25%) was obtained as a red solid after purification by silica gel column chromatography (CH_2Cl_2/MeOH 98:2, R_f (CH_2Cl_2/MeOH 95:5): 0.30). Mp: 135 – 136 ºC; CD ($CHCl_3$): 309 (-0.63); IR (neat): 3333 (w), 2930 (m), 2862 (w), 1693 (m), 1649 (s), 1547 (m), 1439 (s), 1369 (w), 1313 (s), 1241 (m), 1214 (s), 1157 (w), 1070 (w), 1029 (w), 903 (m), 789 (m), 700 (s), 658 (w); ^{1}H NMR (400 MHz, $CDCl_3$): 8.80 (s, 1H), 8.77 (s, 1H), 7.57 – 7.47 (m, 4H), 7.40 – 7.32 (m, 4H), 7.29 (d, *J* = 7.3 Hz, 2H), 5.81 – 5.67 (m, 2H), 5.10 – 4.98 (m, 1H), 4.42 (s, 4H), 3.75 – 3.58 (m, 1H), 3.36 – 3.17 (m, 2H), 2.58 – 2.19 (m, 2H), 2.11 (s, 6H), 2.04 – 1.92 (m, 1H), 1.91 – 1.77 (m, 3H), 1.53 – 1.44 (m, 3H), 1.43 – 1.34 (m, 2H), 1.33 – 1.15 (m, 8 H), 0.98 (d, *J* = 6.5 Hz, 3H), 0.92 (d, *J* = 6.8 Hz, 3H), 0.88 –

0.83 (m, 3H); ^{13}C NMR (100 MHz, $CDCl_3$): 169.0 (C), 164.2 (C), 163.4 (C), 162.6 (C), 162.5 (C), 148.5 (C), 147.9 (C), 147.3 (C), 134.8 (C), 129.6 (CH), 129.5 (CH), 129.4 (CH), 128.9 (CH), 128.8 (CH), 127.8 (CH), 125.2 (C), 124.3 (C), 123.5 (CH), 123.3 (C), 120.6 (CH), 119.7 (C), 118.7 (C), 118.6 (CH), 61.6 (CH), 56.2 (CH), 56.0 (CH), 40.5 (2 x CH_3), 40.0 (CH_2), 37.7 (CH_2), 37.6 (CH_2), 37.4 (CH_2), 31.5 (CH_2), 29.5 (CH_2), 26.9 (CH_2), 26.5 (CH_2), 26.2 (CH_2), 25.8 (CH), 23.3 (CH_3), 23.2 (CH_2), 22.5 (CH_2), 22.2 (CH_3), 14.0 (CH_3); MS (ESI, $CHCl_3$/MeOH (1:1) with 0.1% HCOOH): 833 (100, $[M+H]^+$); HRMS (ESI, +ve) calcd for $C_{48}H_{56}N_4O_5S_2$ ($[M+H]^+$): 833.3765, found: 833.3745.

Compound 7. Following the general procedure **A**, using commercially available dianhydride **24** (250 mg, 0.933 mmol) instead of **23**, pure **7** (181 mg, 33%) was obtained as a red solid after purification by silica gel column chromatography (CH_2Cl_2/MeOH 97:3, R_f (CH_2Cl_2/MeOH 95:5): 0.40). Mp: decomp. 120 – 121 ºC; CD ($CHCl_3$): 381 (+9.60), 360 (+11.89), 338 (+12.83), 281 (-5.91), 258 (-11.43); IR (neat): 3319 (w), 3067 (w), 2929 (m), 2861 (w), 2779 (w), 1696 (m), 1649 (s), 1547 (m), 1436 (s), 1370 (w), 1310 (s), 1268 (w), 1234 (s), 1214 (s), 1022 (w), 905 (w), 789 (w), 750 (m), 691(w); ^{1}H NMR (400 MHz, $CDCl_3$): 8.80 – 8.66 (m, 4H), 5.79 (t, J = 5.7 Hz, 1H), 5.73 (dd, J = 10.0, 5.1 Hz, 1H), 5.08 – 4.92 (m, 1H), 3.71 – 3.58 (m, 1H), 3.38 – 3.20 (m, 2H), 2.54 – 2.40 (m, 1H), 2.39 – 2.27 (m, 1H), 2.13 (s, 6H), 2.02 – 1.92 (m, 2H), 1.91 – 1.79 (m, 2H), 1.66 (brs, 1H), 1.58 – 1.37 (m, 5H), 1.35 – 1.18 (m, 7H), 0.99 (d, J = 6.5 Hz, 3H), 0.93 (d, J = 6.6 Hz, 3H), 0.90 – 0.83 (m, 3H); ^{13}C NMR (100 MHz, $CDCl_3$): 169.0 (C), 163.6 (C), 163.2 (C), 163.1 (C), 162.9 (C), 131.4 (CH), 131.3 (CH), 131.0 (CH), 130.4 (CH), 128.0 (C), 127.1 (C), 126.9 (C), 126.8 (C), 126.1 (C), 125.9 (C), 61.8 (CH), 55.8 (CH), 53.9 (CH), 40.5 (2 x CH_3), 40.0 (CH_2), 37.7 (CH_2), 34.1 (CH_2), 31.4 (CH_2), 29.5 (CH_2), 26.5 (CH_2), 26.1 (CH_2), 25.8 (CH), 25.3 (CH_2), 23.4 (CH_3), 23.0 (CH_2), 22.5 (CH_2), 22.0 (CH_2), 14.0 (CH_3); MS (ESI, $CHCl_3$/MeOH (1:1) with 0.1% HCOOH): 589 (100, $[M+H]^+$); HRMS (ESI, +ve) calcd for $C_{34}H_{45}N_4O_5$ ($[M+H]^+$): 589.3385, found: 589.3376.

Compound 8. Following the general procedure **A**, using dianhydride **22** (200 mg, 0.515 mmol) and **25** (45 mg, 0.52 mmol) instead of **23** and **21**, respectively, pure **8** (126 mg, 41%) was obtained as a red solid after purification by silica gel column chromatography (CH_2Cl_2/MeOH 97:3, R_f (CH_2Cl_2/MeOH 95:5): 0.40). Mp: decomp. 150 – 151 ºC; CD ($CHCl_3$): 429 (-0.27), 356 (+0.56), 257 (-1.73); IR (neat): 2927 (m), 2859 (w), 2824 (w), 1690 (m), 1646 (s), 1548 (m), 1441 (s), 1370 (w), 1313 (s), 1239 (w), 1213 (s), 1192 (m), 1044 (w), 993 (w), 899 (w), 872 (w), 787 (m), 762 (w), 729 (w); ^{1}H NMR (400 MHz, $CDCl_3$): 8.76 – 8.49 (m, 2H), 5.14 – 4.70 (m, 1H), 4.28 – 4.03 (m, 2H), 3.80 – 3.48 (m, 1H), 3.17 (q, J = 7.5 Hz, 4H), 2.60 – 2.27 (m, 1H), 2.08 (s, 6H), 1.96 – 1.61 (m, 7H), 1.46 (t, J = 7.5 Hz, 6H), 1.41 – 1.31 (m, 4H), 1.30 – 1.24 (m, 4H), 0.82 (t, J = 7.0 Hz, 3H); ^{13}C NMR (100 MHz, $CDCl_3$): 163.5 (C), 162.5 (C), 148.4 (C), 147.7 (C), 128.3 (CH), 128.1 (CH), 127.7 (C), 125.0 (C), 124.9 (C), 124.1 (C), 123.6 (C), 120.4 (C), 119.6 (C), 118.7 (C), 61.8 (CH), 55.6 (CH), 41.0 (2 x CH_2), 40.5 (CH_3), 31.5 (CH_2), 29.4 (CH_2), 28.0 (CH_2), 26.8 (CH_2), 26.4 (CH_2), 26.3 (CH_2), 26.2 (CH_2), 25.2 (CH_2), 23.1 (CH_2), 22.6 (CH_2), 14.1 (CH_3), 12.9 (CH_3), 12.8 (CH_3); MS (ESI, $CHCl_3$/MeOH (1:1) with 0.1% HCOOH): 596 (100, $[M+H]^+$); HRMS (ESI, +ve) calcd for $C_{32}H_{41}N_3O_4S_2$ ($[M+H]^+$): 596.2611, found: 596.2619.

Compound 9. Following the general procedure **A**, using dianhydride **22** (165 mg, 0.425 mmol) and **26** (50 mg, 0.43 mmol) instead of **23** and **21**, pure **9** (78 mg, 30%) was obtained as a red solid after purification by silica gel column chromatography (CH_2Cl_2/MeOH 97:3, R_f (CH_2Cl_2/MeOH 95:5): 0.40). Mp: decomp. 137 – 138 ºC; CD ($CHCl_3$): 518 (+0.66), 371 (+0.99), 296 (+0.60); IR (neat): 2929 (m), 2866 (w), 2774 (w), 1692 (m), 1645 (s), 1547 (m), 1440 (s), 1384 (w), 1313 (s), 1260 (w), 1213 (s), 1044 (m), 916 (w), 871 (w), 845 (w), 787 (m), 763 (w), 734 (w), 630 (w); ^{1}H NMR (400 MHz, $CDCl_3$): 8.65 – 8.55 (m, 2H), 5.50 – 5.31 (m, 1H), 5.10 – 4.85 (m, 1H), 4.17 (brs, 1H), 3.88 (dd, J = 11.8, 3.8 Hz, 1H), 3.73 – 3.51 (m, 1H), 3.17 (q, J = 7.4 Hz, 4H), 2.53 – 2.26 (m, 1H), 2.07 (s, 6H), 2.01 – 1.86 (m, 2H), 1.87 – 1.68 (m, 4H), 1.58 – 1.49 (m, 1H), 1.47 (t, J = 7.4 Hz, 6H), 1.40 – 1.27 (m, 2H), 1.26 – 1.12 (m, 2H), 0.88 (t, J = 6.8 Hz, 6H); ^{13}C NMR (100 MHz, $CDCl_3$): 164.5 (C), 163.8 (C), 163.2

(C), 162.6 (C), 148.3 (C), 147.7 (C), 128.3 (CH), 127.7 (CH), 125.1 (C), 125.0 (C), 124.2 (C), 120.5 (C), 119.7 (C), 63.8 (CH_2), 61.8 (CH), 56.2 (CH), 55.6 (CH), 54.5 (CH), 40.5 (2 x CH_3), 37.4 (CH_2), 29.5 (CH_2), 29.4 (CH_2), 26.4 (CH_2), 26.3 (CH_2), 25.5 (CH), 25.2 (CH_2), 23.1 (CH_2), 22.9 (CH_3), 22.7 (CH_3), 12.9 (CH_3), 12.8 (CH_3); MS (ESI, $CHCl_3$/MeOH (1:1) with 0.1% HCOOH): 612 (100, $[M+H]^+$); HRMS (ESI, +ve) calcd for $C_{32}H_{41}N_3O_5$ ($[M+H]^+$): 612.2560, found: 612.2560.

Compound 13. Following the general procedure **A**, starting from dianhydride **24** (125 mg, 0.466 mmol), quinine amine (**27**, 151 mg, 0.466 mmol) and L-leucine derivative (**21**, 120 mg, 0.466 mmol), pure compound **23** (125 mg, 35%) was obtained as a red solid after purification by silica gel column chromatography (CH_2Cl_2/MeOH 97:3, R_f (CH_2Cl_2/MeOH 95:5): 0.40). Mp: 139 – 140 ºC; CD ($CHCl_3$): 384 (-2.75), 370 (-1.76), 360 (-3.34), 344 (-2.21), 327 (+2.54); IR (neat): 3321 (w), 2928 (m), 2862 (w), 1705 (m), 1656 (s), 1547 (m), 1437 (m), 1315 (s), 1233 (s), 1217 (s), 1190 (m), 1024 (w), 827 (w), 755 (m), 692 (w); ^{1}H NMR (400 MHz, $CDCl_3$): 8.89 (d, *J* = 4.7 Hz, 1H), 8.85 (d, *J* = 7.6 Hz, 1H), 8.76 (d, *J* = 7.6 Hz, 1H), 8.67 (d, *J* = 7.7 Hz, 1H), 8.56 (d, *J* = 7.7 Hz, 1H), 8.01 (d, *J* = 9.2 Hz, 1H), 7.95 (d, *J* = 4.7 Hz, 1H), 7.80 (d, *J* = 2.5 Hz, 1H), 7.32 (dd, *J* = 9.2, 2.5 Hz, 1H), 6.84 (d, *J* = 11.1 Hz, 1H), 6.15 – 6.01 (m, 1H), 5.78 (t, *J* = 5.5 Hz, 1H), 5.72 (dd, *J* = 10.0, 5.1 Hz, 1H), 5.18 (s, 1H), 5.14 (d, *J* = 7.0 Hz, 1H), 4.65 (q, *J* = 9.1 Hz, 1H), 3.99 (s, 3H), 3.48 – 3.36 (m, 1H), 3.36 – 3.22 (m, 2H), 3.09 (dd, *J* = 13.7, 10.1 Hz, 1H), 2.83 (d, *J* = 13.7 Hz, 1H), 2.72 – 2.58 (m, 1H), 2.40 – 2.26 (m, 2H), 2.20 – 2.09 (m, 1H), 2.00 – 1.88 (m, 1H), 1.83 (brs, 1H), 1.74 – 1.62 (m, 4H), 1.61 – 1.39 (m, 4H), 1.38 – 1.22 (m, 7H), 0.98 (d, *J* = 6.5 Hz, 3H), 0.93 (d, *J* = 6.5 Hz, 3H), 0.91 – 0.84 (m, 3H); ^{13}C NMR (100 MHz, $CDCl_3$): 168.9 (C), 163.6 (C), 162.9 (C), 162.8 (C), 162.7 (C), 158.4 (C), 147.0 (CH), 145.0 (C), 142.1 (C), 138.9 (C), 131.9 (CH), 131.4 (CH), 131.3 (CH), 131.2 (CH), 130.9 (CH), 129.5 (C), 127.4 (C), 126.9 (C), 126.8 (C), 126.7 (C), 126.4 (C), 126.1 (C), 124.3 (CH), 122.0 (CH), 114.6 (CH_2), 101.5 (C), 56.3 (CH_2), 55.7 (CH_3), 54.0 (CH), 53.9 (CH), 53.8 (CH), 41.9 (CH_2), 40.0 (CH_2), 39.9 (CH), 37.6 (CH_2), 31.4(CH_2), 29.4(CH_2),

28.9 (CH_2), 27.9 (CH_2), 27.7 (CH), 26.5 (CH_2), 25.7 (CH), 23.3 (CH_3), 22.5 (CH_2), 22.0 (CH_3), 14.0 (CH_3); MS (ESI, $CHCl_3$/MeOH (1:1) with 0.1% HCOOH): 770 (100, $[M+H]^+$); HRMS (ESI, +ve) calcd for $C_{46}H_{51}N_5O_6$ ($[M+H]^+$): 770.3932, found: 770.3912.

Compound 14. To a suspension of succinic anhydride (**28**, 41 mg, 0.41 mmol) in toluene (3.0 mL) were added **27** (133 mg, 0.411 mmol) and 4 Å molecular sieves (20 mg) at rt with stirring. The resulting mixture was heated at 110 ^{o}C under argon atmosphere for about 24 h. Then, the mixture was cooled to rt and concentrated *in vacuo*. Silica gel column chromatography of the residue (ethyl acetate/petroleum ether 7:3, R_f (ethyl acetate/petroleum ether 1:1): 0.50) gave pure **14** (110 mg, 70%) as a white solid. Mp: 127 – 128 ºC; CD ($CHCl_3$): 336 (-1.25), 279 (-0.95), 250 (-3.53); IR (neat): 2938 (w), 2863 (w), 1773 (w), 1699 (s), 1620 (m), 1507 (m), 1478 (w), 1433 (w), 1380 (m), 1361 (m), 1228 (s), 1162 (s), 1028 (m), 999 (w), 912 (m), 852 (m), 830 (m), 820 (m), 756 (w), 713 (m), 666 (m), 622 (m); ^1H NMR (400 MHz, $CDCl_3$): 8.78 (d, J = 4.6 Hz, 1H), 8.02 (d, J = 9.2 Hz, 1H), 7.73 (d, J = 4.6 Hz, 1H), 7.69 (d, J = 2.5 Hz, 1H), 7.36 (dd, J = 9.2, 2.5 Hz, 1H), 6.02 – 5.90 (m, 1H), 5.84 (d, J = 11.5 Hz, 1H), 5.16 – 5.03 (m, 2H), 4.35 (q, J = 9.1 Hz, 1H), 3.99 (s, 3H), 3.31 – 3.09 (m, 2H), 2.89 – 2.73 (m, 2H), 2.73 – 2.50 (m, 3H), 2.50 – 2.37 (m, 1H), 2.32 (s, 1H), 1.91 (t, J = 10.9 Hz, 1H), 1.72 (brs, 1H), 1.63 – 1.47 (m, 2H), 0.74 – 0.58 (m, 1H); ^{13}C NMR (100 MHz, $CDCl_3$): 177.6 (C), 177.1 (C), 158.5 (C), 147.2 (CH), 144.9 (C), 141.7 (CH_2), 138.3 (C), 131.9 (CH), 129.0 (C), 122.7 (CH), 122.1 (CH), 114.6 (C), 101.2 (CH), 55.9 (CH_3), 52.7 (CH), 51.4 (CH), 41.1 (CH), 39.5 (CH), 28.0 (CH), 27.6 (CH), 27.5 (CH_2); MS (ESI, $CHCl_3$/MeOH (1:1) with 0.1% HCOOH): 406 (100, $[M+H]^+$); HRMS (ESI, +ve) calcd for $C_{24}H_{27}N_3O_3$ ($[M+H]^+$): 406.2125, found: 406.2113.

3. Catalysis evaluation

3.1. Anion-π catalysts

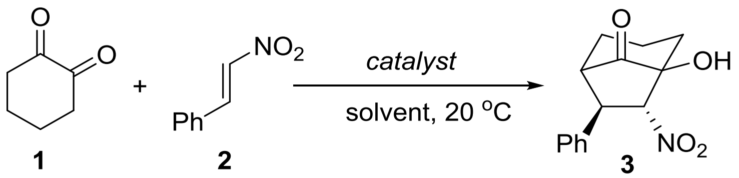

Solutions of substrates **1** (0.4 M) and **2** (0.8 M), internal standard dibromomethane (0.4 M) and catalyst (**4**: 0.04 M, 0.02 M or 0.01 M; **5**, **6**, **7**, **8**, **9**, **13**, **14**, **15**: 0.02 M; **11** + **12**: 0.02 + 0.02 M) were prepared in the corresponding solvents including $CDCl_3$, CD_3CN, THF-d_8, toluene-d_8, benzene-d_6, $C_6F_6/CDCl_3$ (4:1), $C_6F_6/CDCl_3$ (3:1), $C_6F_6/CDCl_3$ (2:1), C_6F_6/CD_3CN (4:1), C_6F_6 and nitrobenzene. The reaction mixture was stirred at 20 °C. ^{1}H NMR spectra of the mixture diluted in $CDCl_3$ were recorded (Figure S1). The concentration of the products **3** was determined by comparing the integration of pertinent resonances with those of internal standards in the crude NMR.

Upon complete consumption of substrate **1** indicated by ^{1}H NMR, typically in less than 72 h, the mixture was concentrated under reduced pressure at rt and the residue was subjected to PTLC purification directly to obtain pure products **3**. The spectroscopic data obtained for products **3** and **3d** were identical to the ones reported in the literature.[S10]

Diastereoselectivity between the major isomers **3** and **3d** was determined from the integrals of pertinent peaks in crude NMR spectra of the reaction mixtures (Figures S2-S7). Enantioselectivity of the major product **3** was analyzed by chiral HPLC (column: Chiralpak AD column; mobile phase: *n*-hexane/*i*-PrOH 90/10, 1.0 mL/min, room temperature; detection: 220 nm, Figures S8-S11). Under these conditions, the product **3** obtained using reported cinchonine catalyst **15** in toluene-d_8 gave a major peak at $R_t \approx 22$ min and minor peak at $R_t \approx 56$ min (Figure 2d). The major peak can be assigned to originate in 1*R*,5*R*,6*S*,7*S*-**3**, as it is reported to be the

major enantiomer by Ding and co-workers.[S11] In contrast, those obtained using NDI catalysts gave the enantiomeric peak at $R_t \approx 56$ min as major peak. Thus, we concluded that the absolute configuration of the isomers obtained using NDI catalysts are the opposite of those with **15**. They are 1*S*,5*S*,6*R*,7*R*-**3** and 1*R*,5*R*,6*S*,7*S*-**3e**.

For the inhibition experiments, solution of substrate **1** (0.4 M), substrate **2** (0.8 M), internal standard dibromomethane (0.4 M), catalyst (**5**: 0.02 M; **11**: 0.04 M + **12**: 0.04 M; **13**: 0.02 M) and the indicated amount of tetrabutylammonium salt (NO_3^-: 0.3 M, 0.6 M, 0.8 M, 1.0 M, 1.2 M or 2.0 M; PF_6^-: 1.2 M; BF_4^-: 1.2 M; Br^-: 1.2 M) were prepared in C_6F_6/$CDCl_3$ (4:1). The reaction mixture was stirred at 20 ^oC. Upon complete consumption of substrate **1** indicated by ^1H NMR, the mixture was concentrated at rt and subjected to PTLC purification. For the ones inhibited by nitrate, the mixtures were stirred at 20 ^oC for 120 h. Then, the mixture was concentrated under reduced pressure at rt and subjected to PTLC purification. Diastereo- and enantioselectivities were determined as described above (Figures S6, S7, S11, S12).

For the kinetic studies, concentrations of product **3** were plotted against time. The initial velocities (v_{ini}) were determined from the linear fitting (Figures 2a-c, S14-16). From equation (S1), we could get the apparent second-order rate constants (k_{app}).

$$k_{app} = v_{ini} /([\mathbf{1}]_0[\mathbf{2}]_0 \qquad \text{(S1)}$$

Then the rate enhancements Δv_{ini} were calculated from equation (S2).

$$\Delta v_{\text{ini}} = k_{app}\,(1) \,/\, k_{app}\,(2) \qquad \text{(S2)}$$

Transition-state stabilizations ΔE_a were determined by equation (S3).

$$\Delta E_{\text{a}} = -\,RT \ln \Delta v_{\text{ini}} \qquad \text{(S3)}$$

The velocities were plotted against nitrate concentration and fitted to Hill equation (S4) to determine the IC_{50} for the nitrate inhibition experiments (Figures S17-S18).

$$
\mathrm{Y} = \frac{1 + \mathrm{Y}_{\infty} \times \left(\frac{[\mathrm{NO_3^-}]}{\mathrm{IC_{50}}}\right)^{n}}{1 + \left(\frac{[\mathrm{NO_3^-}]}{\mathrm{IC_{50}}}\right)^{n}} \qquad \text{(S4)}
$$

3.2. Anion-π enzymes

Stock solutions of substrates **1** (80 mM), **2** (200 mM) and biotinylated catalysts **16** (2 mM) were prepared in CD_3CN. Solutions of substrate **1** should be freshly prepared.

Solutions were prepared by mixing successively streptavidin WT or mutants (200 µL, 1 mM, Bis-Tris pH 6.5, 0.2 µmol), biotinylated ligands **16** (50 µL, 0.1 µmol), substrates **1** (25 µL, 10 µmol) and **2** (25 µL, 25 µmol) and stirred at 20 °C. After given time, ^{1}H NMR of the mixture, extracted with $CDCl_3$ (0.7 mL), dried over Na_2SO_4, and filtered into NMR tube, was recorded.

The spectroscopic data obtained for product **3** were identical to the ones reported in the literature.[S10] Crude mixtures were analyzed by chiral HPLC, (*i.e.*, column: CHIRALPAK AD-H column; mobile phase: *n*-Hexane/*i*-PrOH 90/10, 1.0 mL/min, rt; detection: 220 nm, Figure S13). The wild-type streptavidin and various mutants were expressed and purified according to the previously reported protocol [S12, S13] and screened with catalyst **16** (Table S2)

Inhibition experiments were carried out by preparing solutions of streptavidin S112W (0.66 mM), **8** (0.33 mM) and inhibitor (0.05-1.0 M) followed by **1** and **2**. Reactions were monitored by ^{1}H NMR spectroscopy. Inhibition concentration IC_{50} and Hill coefficients n were determined by plotting the *ee* after completion of the reaction as a function of NO_3^- concentration c and fitting them to the Hill equation (S5)

$$
Y = ee_0 + (ee_{min} - ee_0) / \{1 + (IC_{50} / c)^n\} \qquad \text{(S5)}
$$

where ee_0 is the *ee* without NO_3^-, ee_{min} is the *ee* at NO_3^- saturation, IC_{50} is the concentration of NO_3^- required to inhibit 50% of the decrease in stereoselectivity and n is the Hill coefficient (Figure S19).

4. Supplementary figures and tables

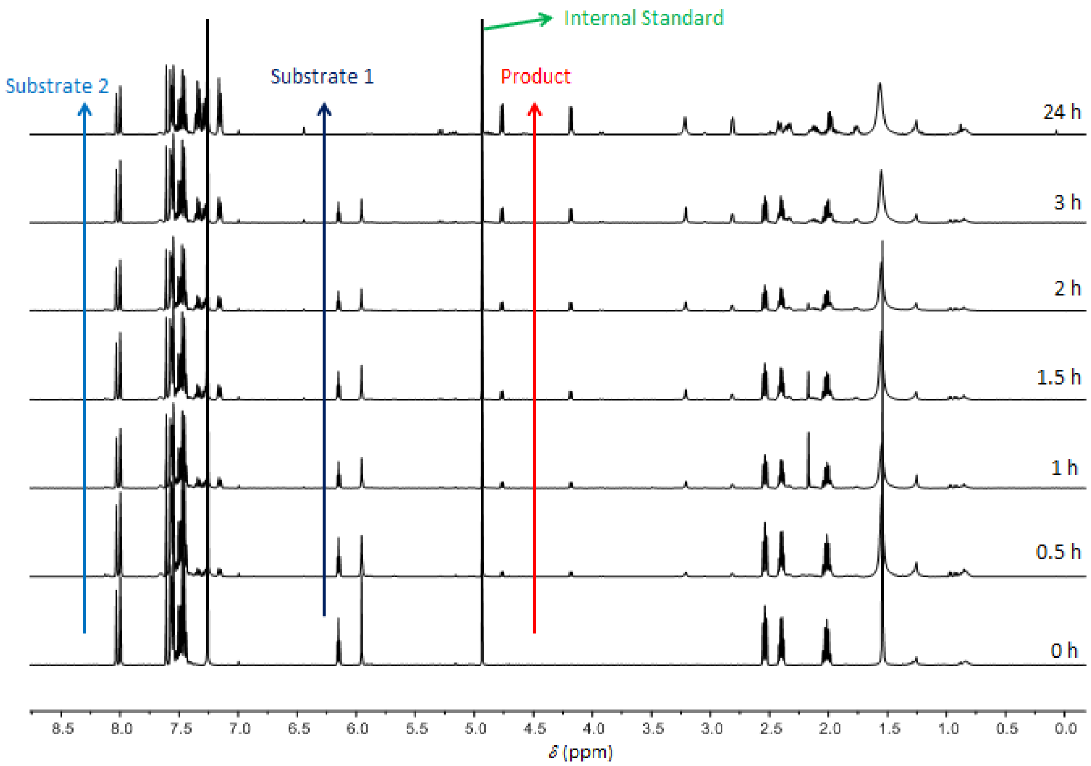

Figure S1. ^{1}H NMR spectra of a mixture of cyclohexane-1,2-dione **1** (0.4 M), (*E*)-(2-nitrovinyl)benzene **2** (0.8 M) and catalyst **5** (0.02 M) in C_6F_6 at 20 ^{o}C diluted in $CDCl_3$. The blue arrows show the consumption of cyclohexane-1,2-dione **1** and (*E*)-(2-nitrovinyl)benzene **2**. The red one shows the formation of the product. Dibromomethane (0.4 M) is used as an internal standard (green).

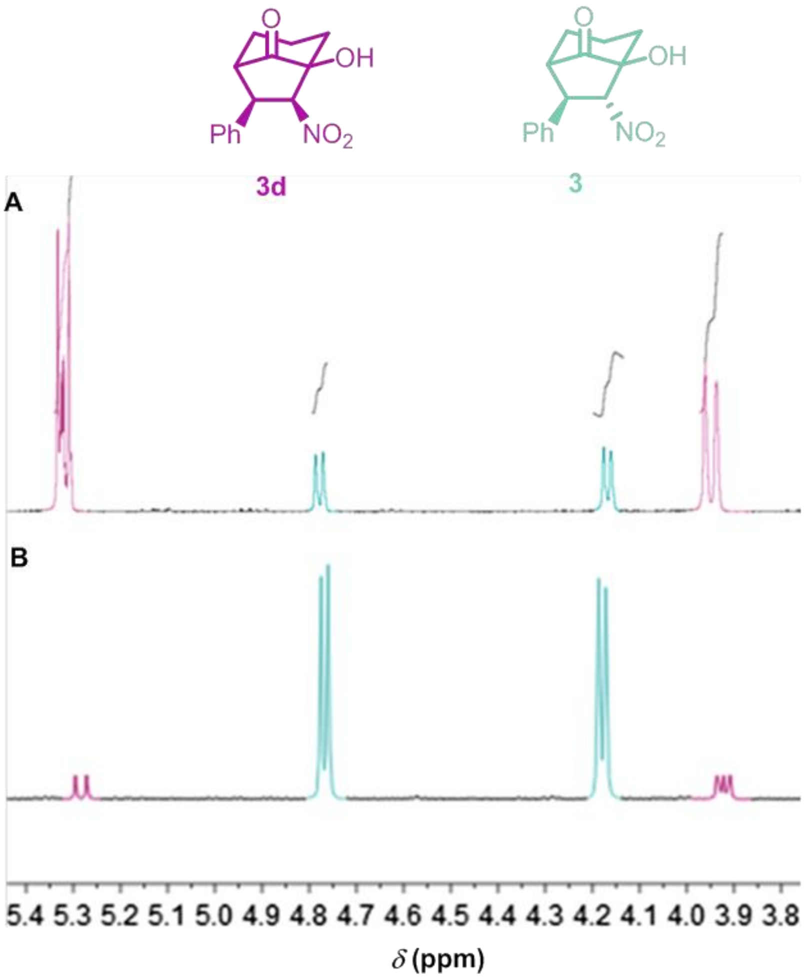

Figure S2. Comparison of the diagnostic regions of ^{1}H NMR spectra of product **3** (**A**: reported spectrum by Rueping and co-workers;[S10] **B**: spectrum of the product obtained using NDI catalysts).

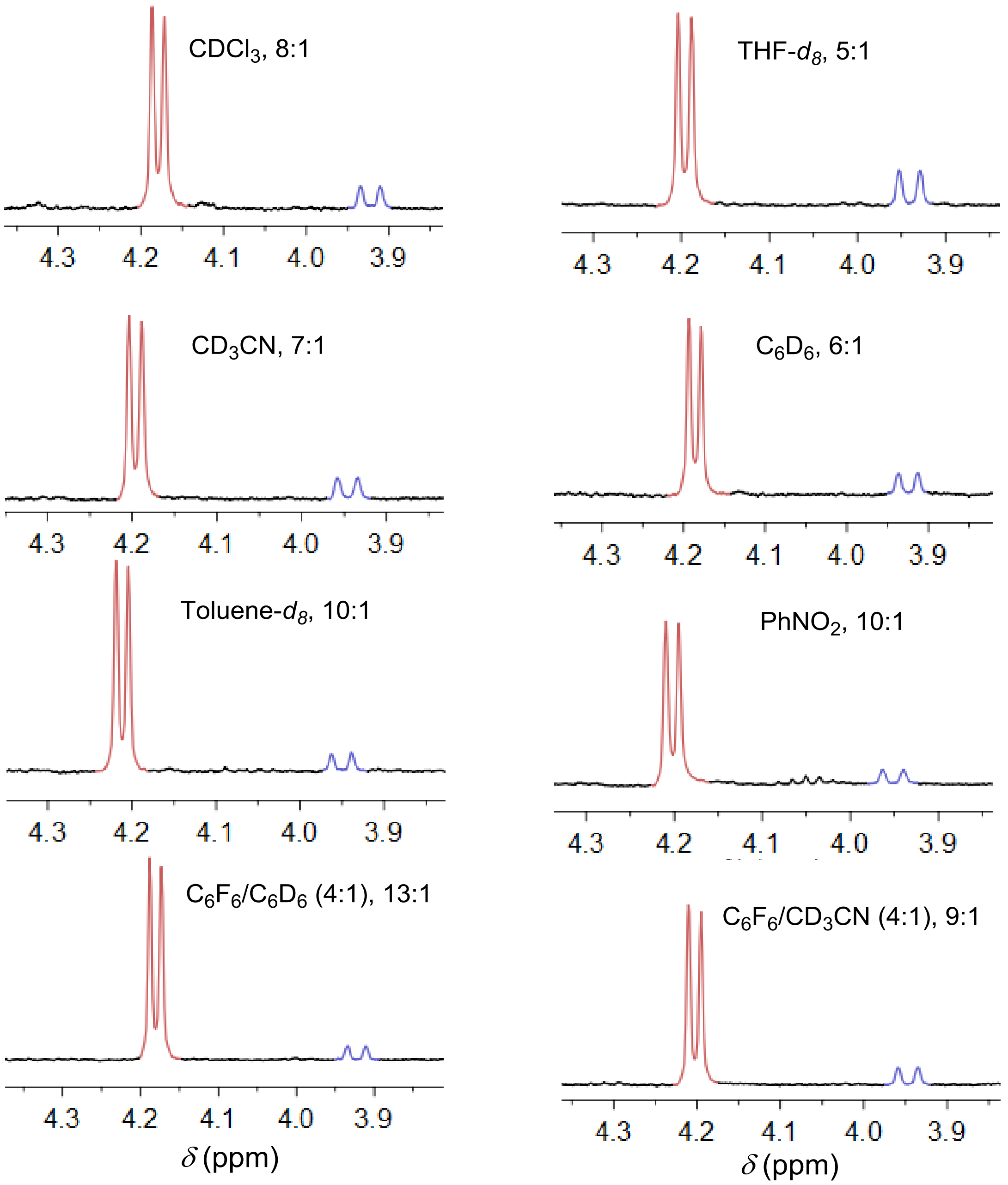

Figure S3. Diagnostic regions of crude ^{1}H NMR spectra of the reaction mixture used to determine the diastereomeric ratio of **3** (red) and **3d** (blue) catalyzed by **4** (10 mol%) in the indicated solvent.

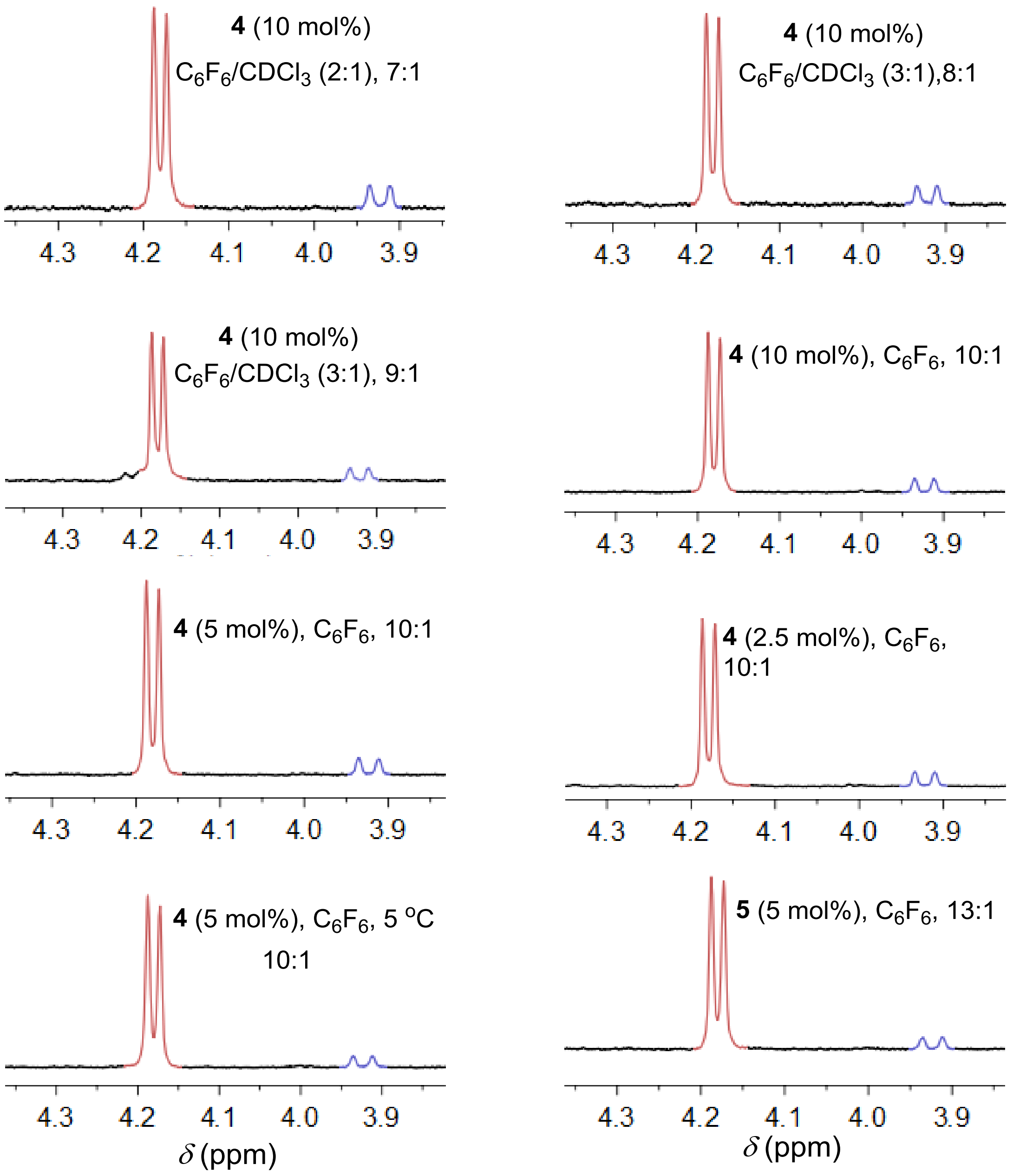

Figure S4. Diagnostic regions of crude ^{1}H NMR spectra of the reaction mixture used to determine the diastereomeric ratio of **3** (red) and **3d** (blue) under the indicated conditions.

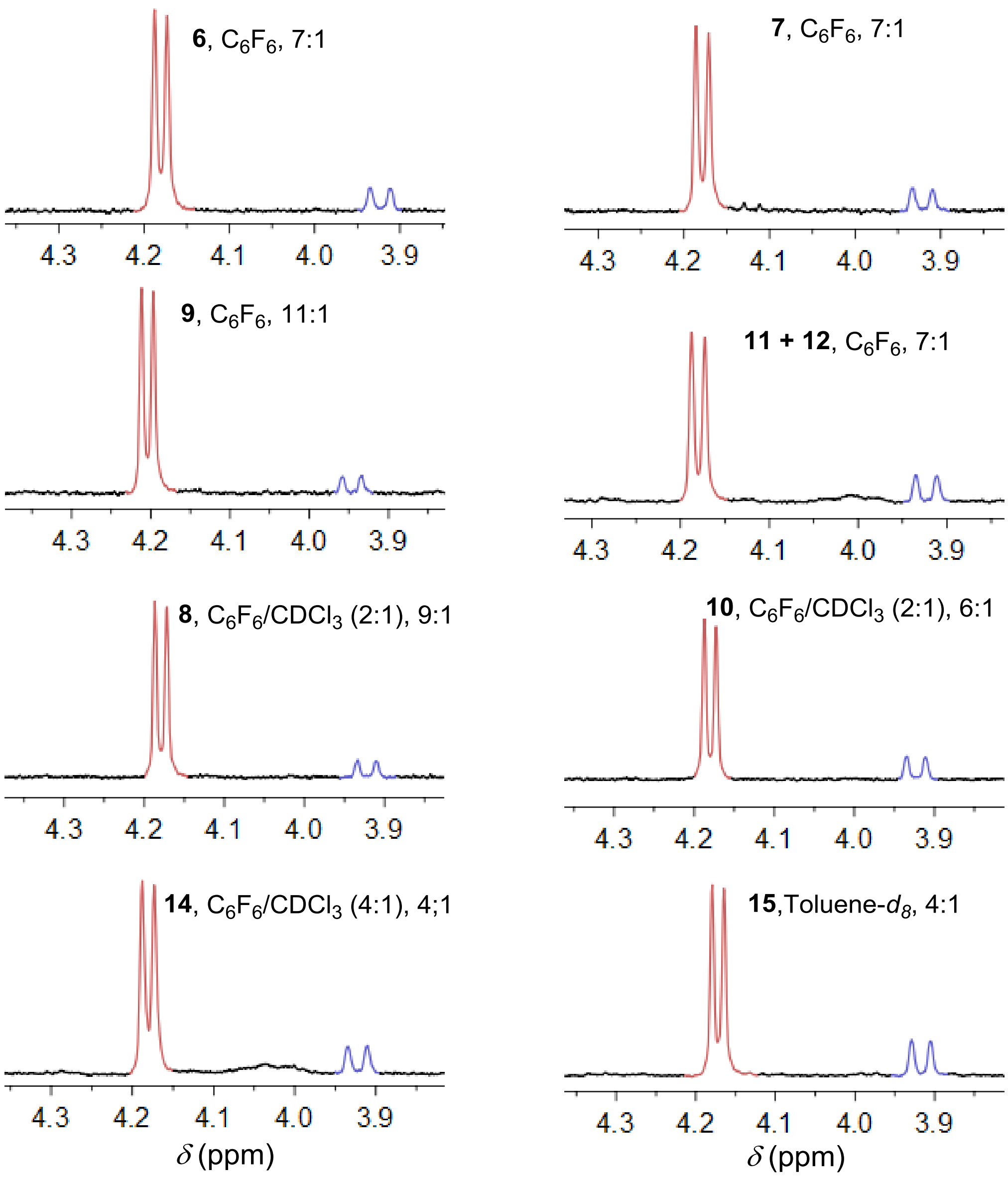

Figure S5. Diagnostic regions of crude ^{1}H NMR spectra of the reaction mixture used to determine the diastereomeric ratio of **3** (red) and **3d** (blue) catalyzed by 5 mol% of the corresponding catalyst under indicated conditions.

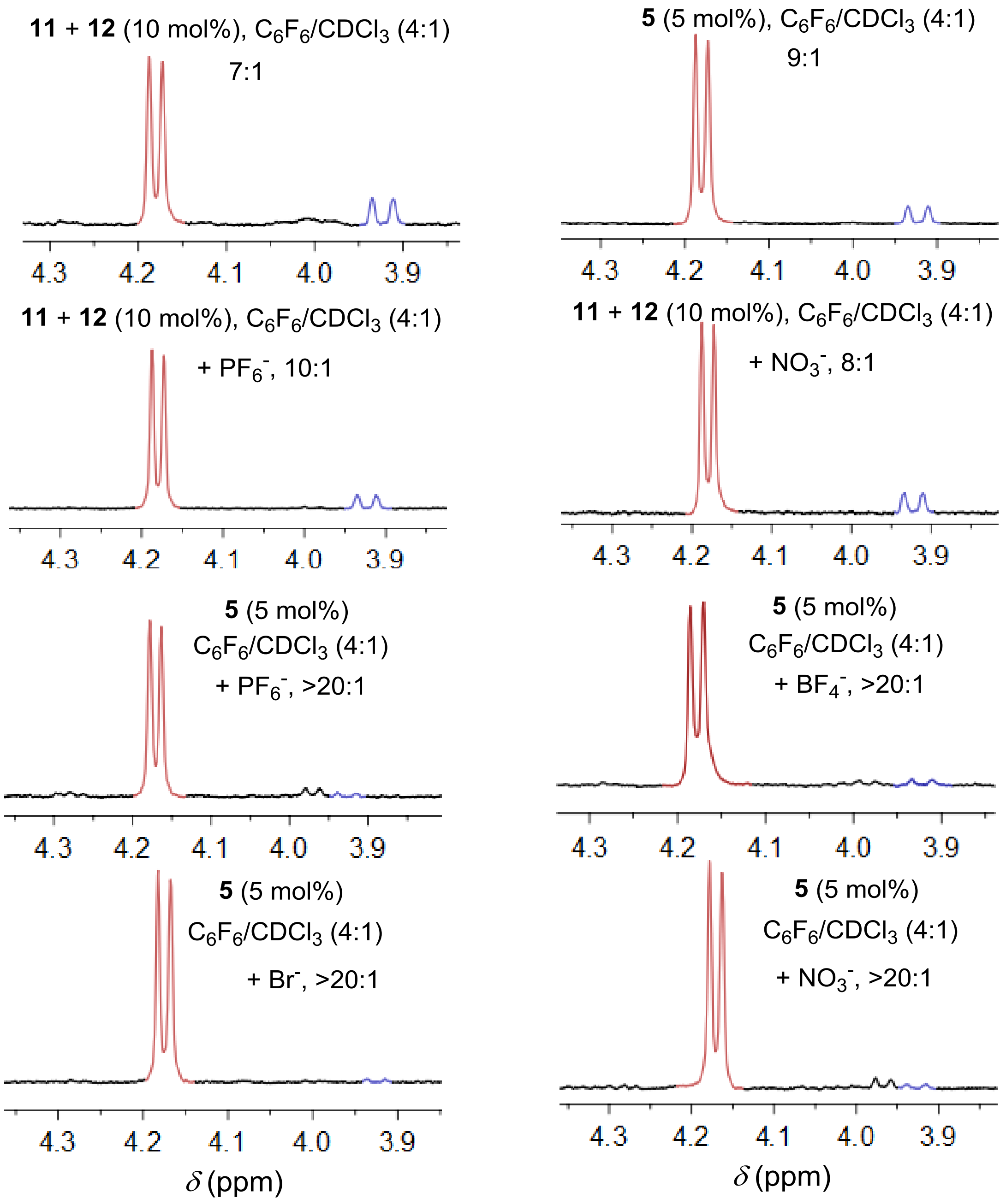

Figure S6. Diagnostic regions of crude ^{1}H NMR spectra of the reaction mixture used to determine the diastereomeric ratio of **3** (red) and **3d** (blue) under the indicated conditions.

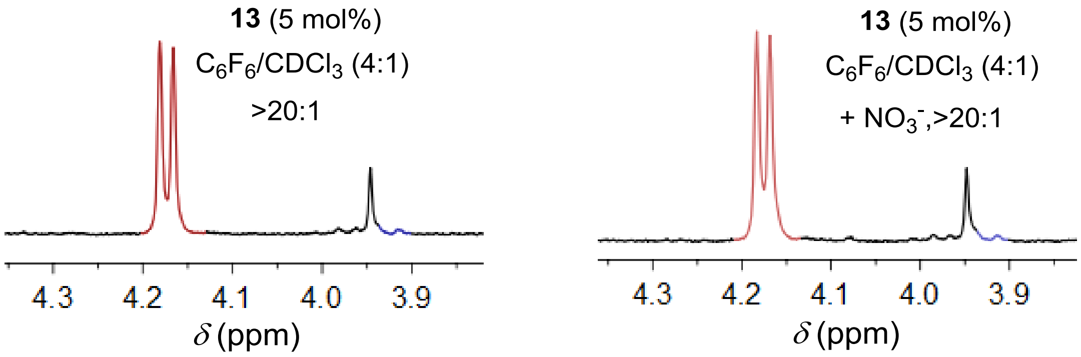

Figure S7. Diagnostic regions of crude ^{1}H NMR spectra of the reaction mixture used to determine the diastereomeric ratio of **3** (red) and **3d** (blue) under the indicated conditions.

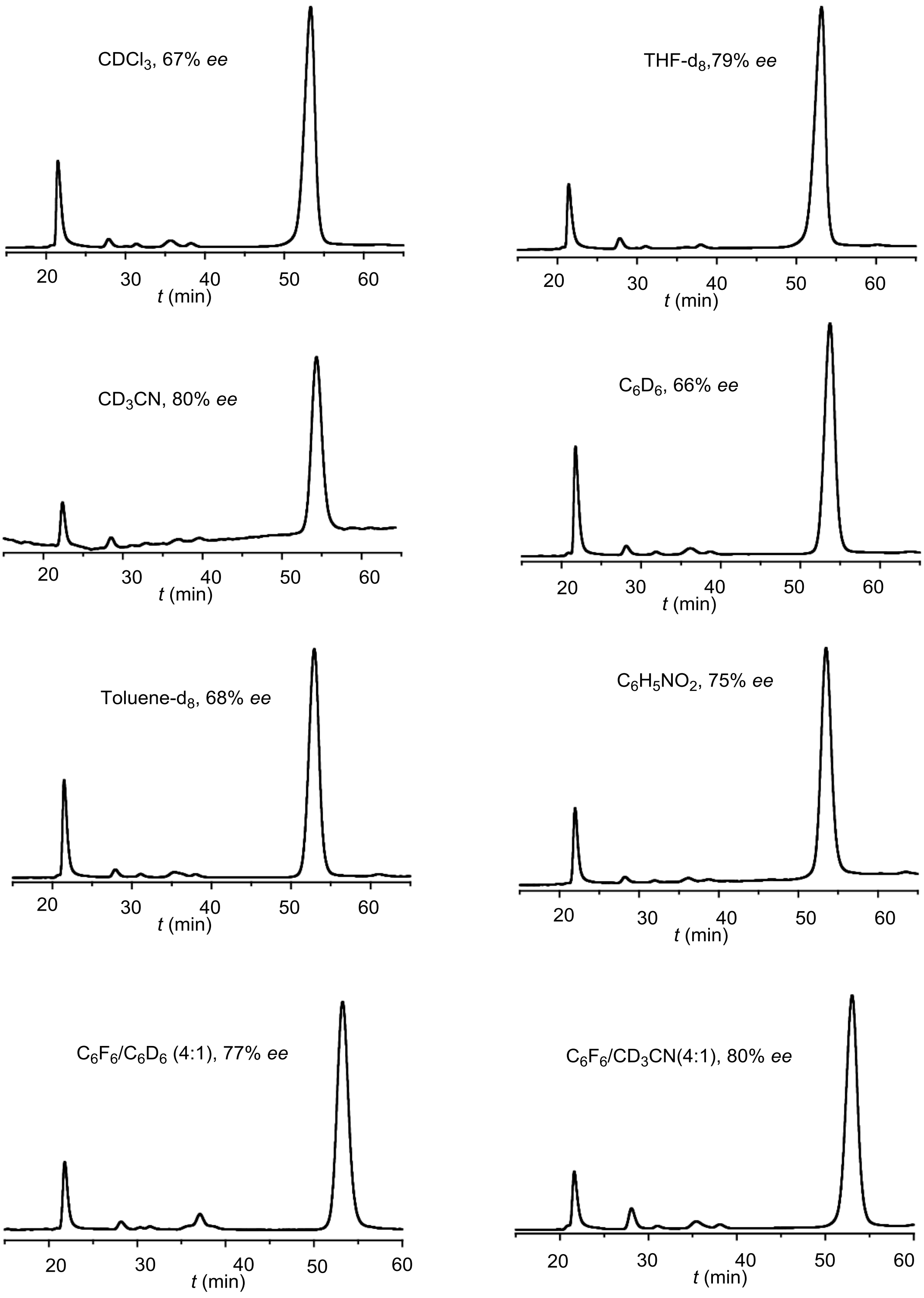

Figure S8. Chiral HPLC traces of **3** obtained using Chiralpak AD column (*n*-hexane/*i*-PrOH 90:10, rt, 1.0 mL/min, 220 nm) for the reactions catalyzed by **4** (10 mol%) in the indicated solvent.

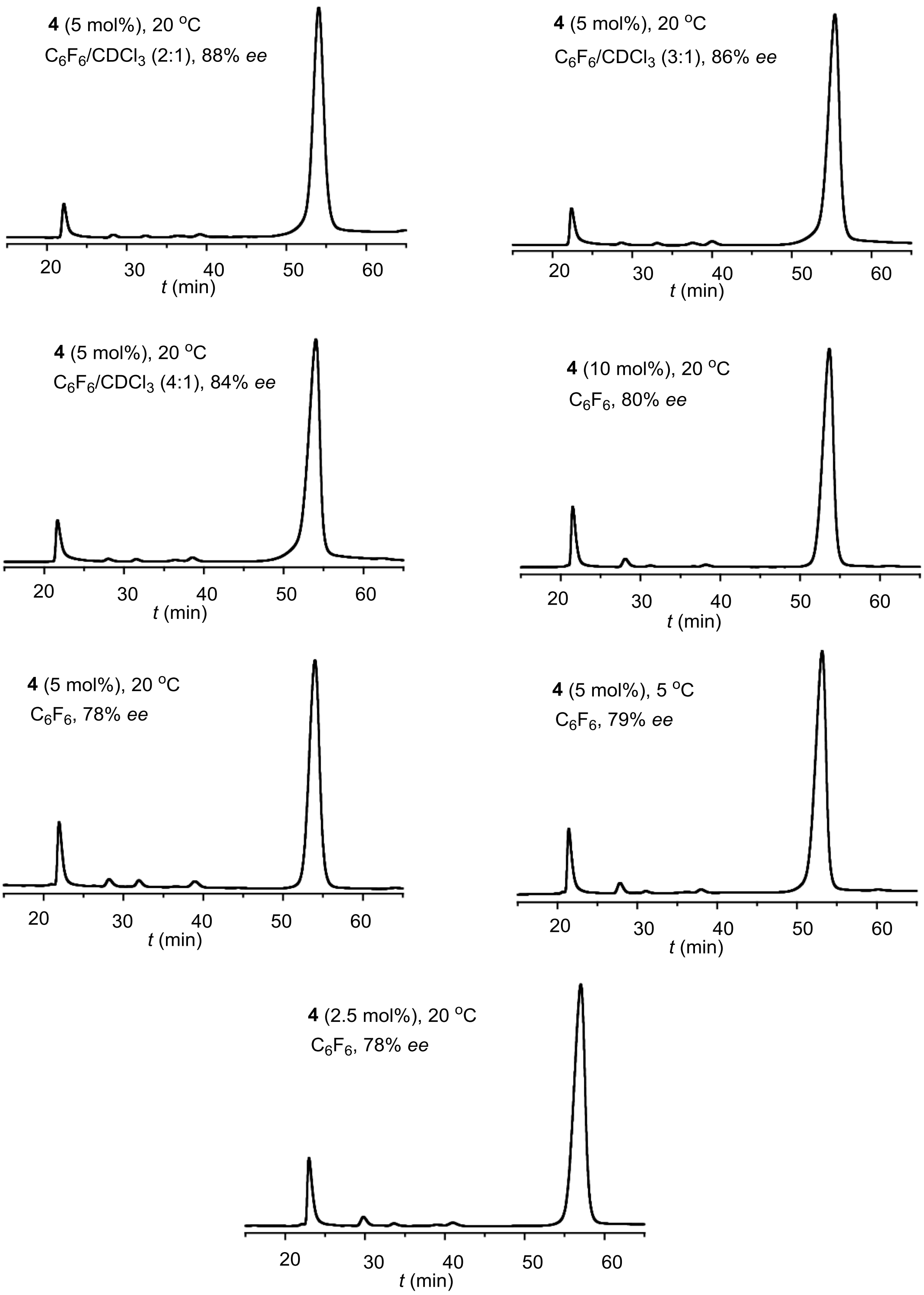

Figure S9. Chiral HPLC traces of **3** obtained using Chiralpak AD column (*n*-hexane/*i*-PrOH 90:10, rt, 1.0 mL/min, 220 nm) for the reactions catalyzed by **4** under the indicated conditions.

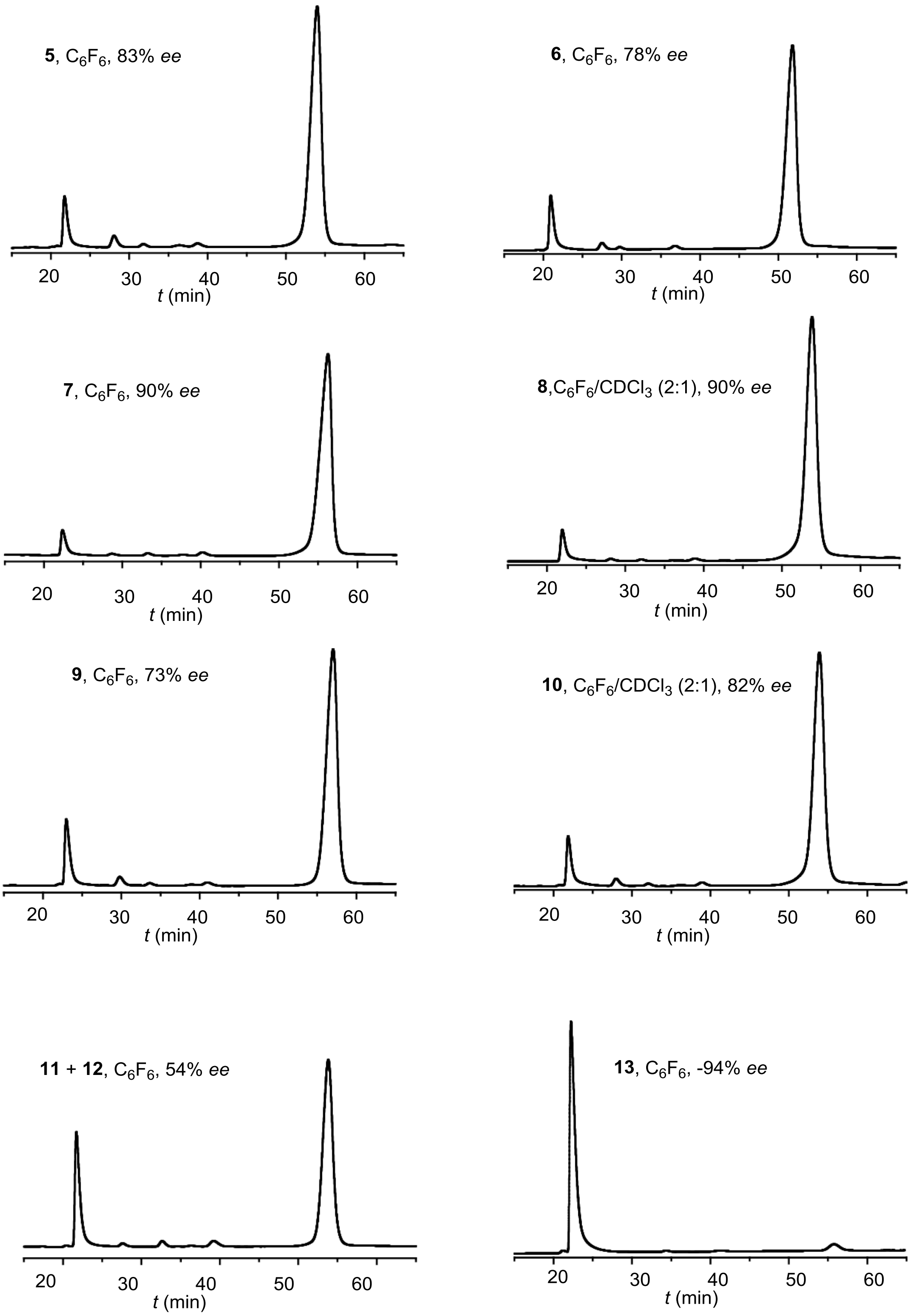

Figure S10. Chiral HPLC traces of **3** obtained using Chiralpak AD column (*n*-hexane/*i*-PrOH 90:10, rt, 1.0 mL/min, 220 nm) for the reactions catalyzed by different catalyst (5 mol%) under the indicated conditions.

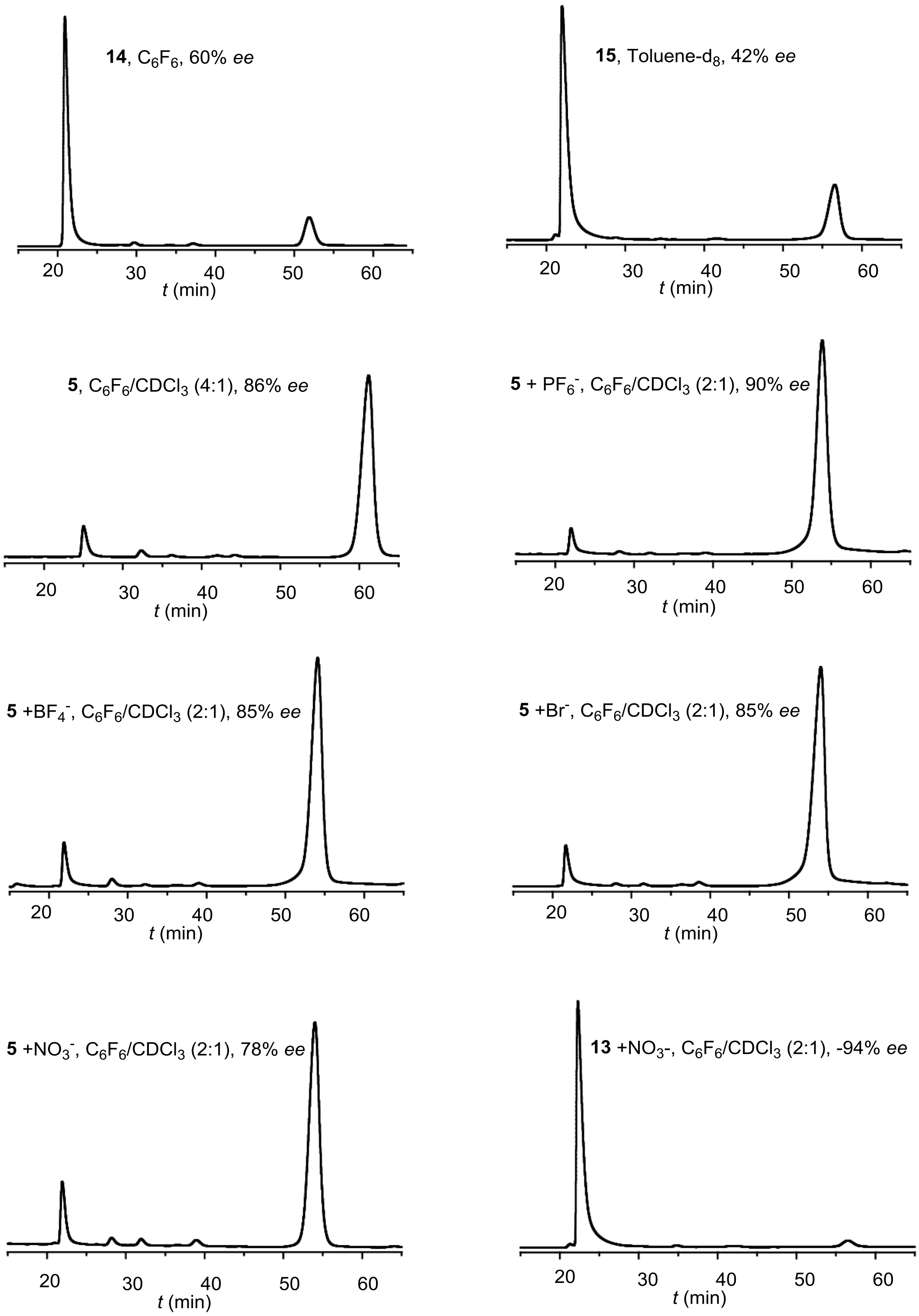

Figure S11. Chiral HPLC traces of **3** obtained using Chiralpak AD column (*n*-hexane/*i*-PrOH 90:10, rt, 1.0 mL/min, 220 nm) for the reactions catalyzed by different catalyst (5 mol%) under the indicated conditions.

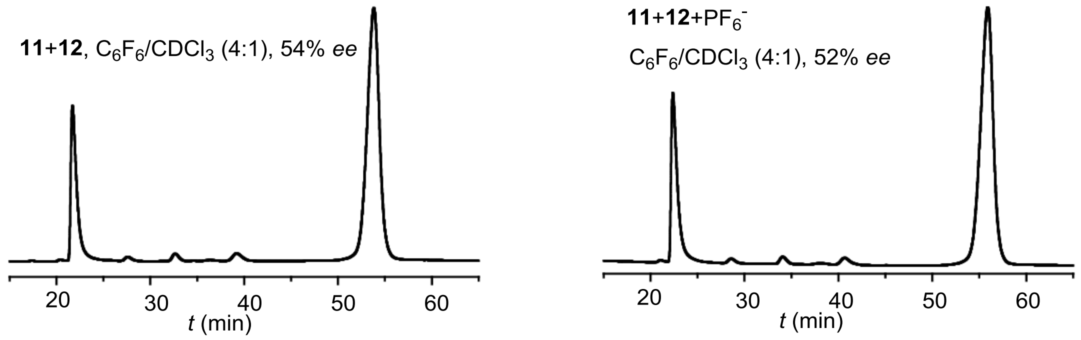

Figure S12. Chiral HPLC traces of **3** obtained using Chiralpak AD column (*n*-hexane/*i*-PrOH 90:10, rt, 1.0 mL/min, 220 nm) for the reactions catalyzed by catalyst (10 mol%) under the indicated conditions.

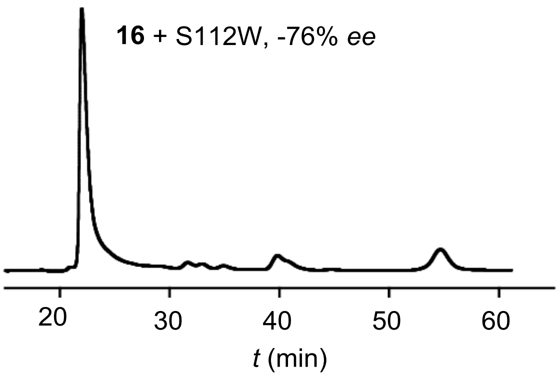

Figure S13. Chiral HPLC traces of **3** obtained using Chiralpak AD column (*n*-hexane/*i*-PrOH 90:10, rt, 1.0 mL/min, 220 nm) for the reactions catalyzed by **16** + S112W.

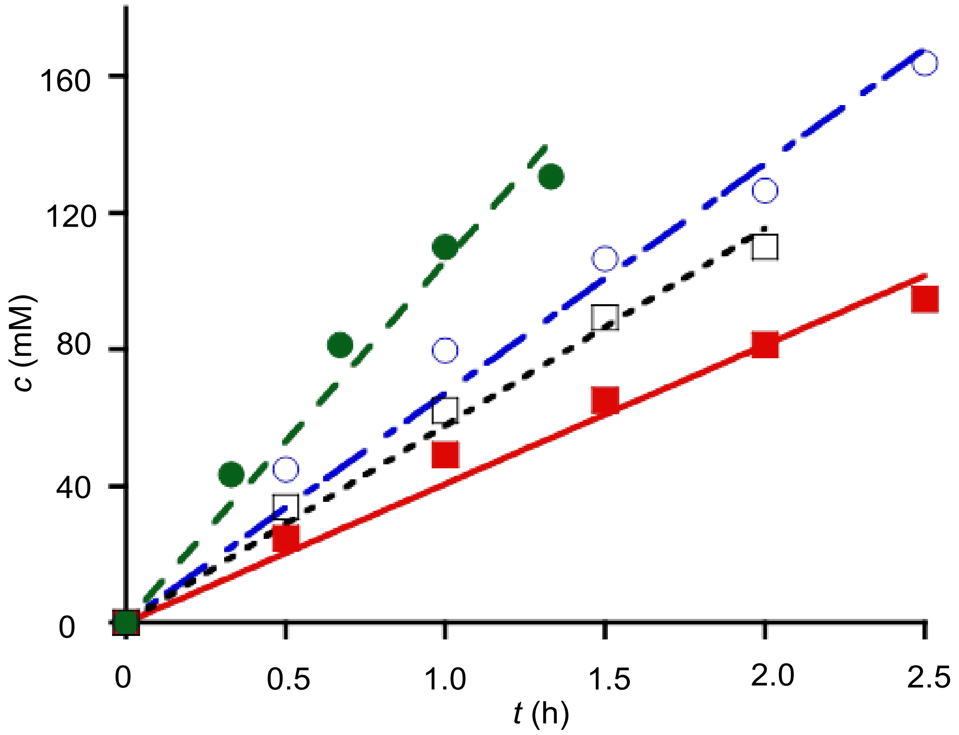

Figure S14. Kinetics for the reaction catalyzed by **10** (5 mol%) (green filled circles), **10** (5 mol%) + PivOH (5 mlo%) (blue empty circles), **4** (5 mol%) (black empty circles) and **10** (2.5 mol%) (filled red squares) in $C_6F_6/CDCl_3$ (2:1) at 20 ^{o}C. The initial velocity was determined by the linear fitting.

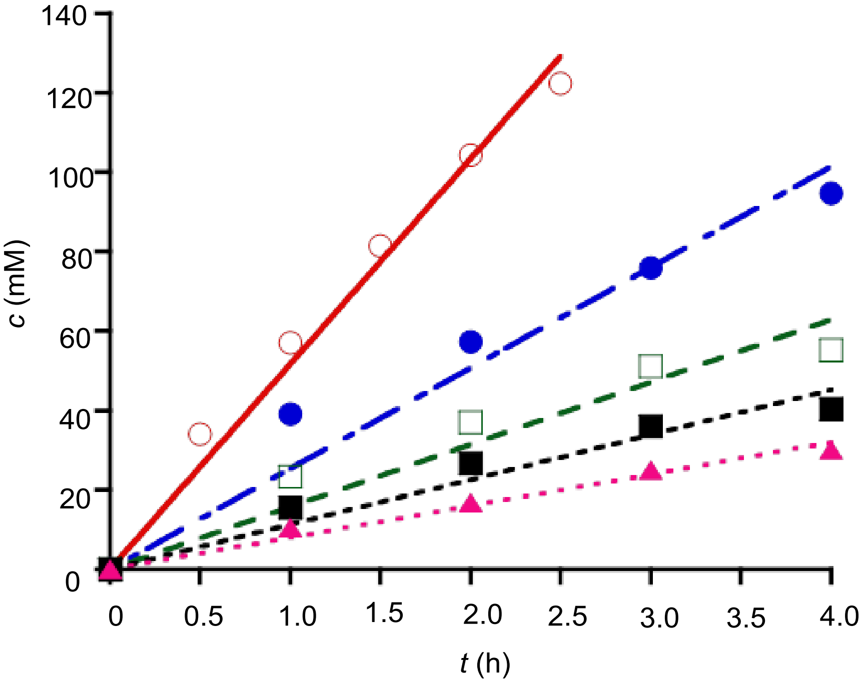

Figure S15. Kinetics for the reaction catalyzed by 5 mol% **13** (red empty circles) and reactions which are catalyzed by **13** in the presence of different concentration of $TBANO_3$(0.8 M (blue filled circles), 1.2 M (green empty squares), 1.6 M (black filled squares) and 2.0 M (pink filled triangles)) in $C_6F_6/CDCl_3$ (4:1) at 20 °C. The initial velocity was determined by the linear fitting.

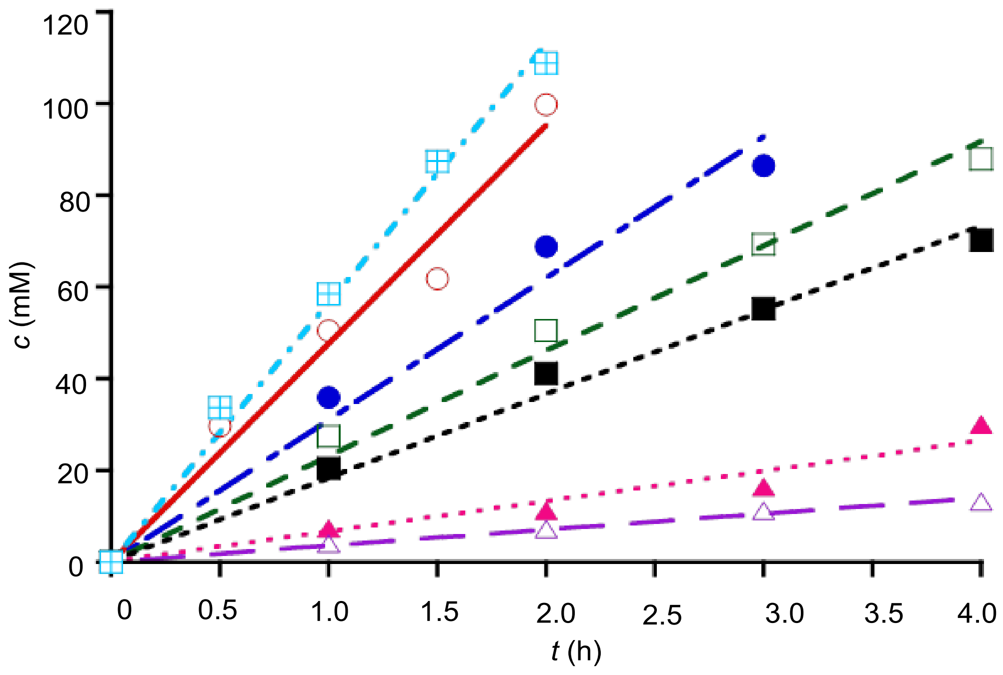

Figure S16. Kinetics for the reaction catalyzed by **5** (red empty circles) and reactions which are catalyzed by **5** in the presence of different concentration of $TBANO_3$ (0.3 M (turquoise squared croses), 0.6 M (blue filled circles), 0.8 M (green empty squares), 1.0 M (black filled squares), 1.2 M (pink filled triangles) and 2.0 M (purple empty triangles)) in $C_6F_6/CDCl_3$ (4:1) at 20 ^{o}C. The initial velocity was determined by the linear fitting.

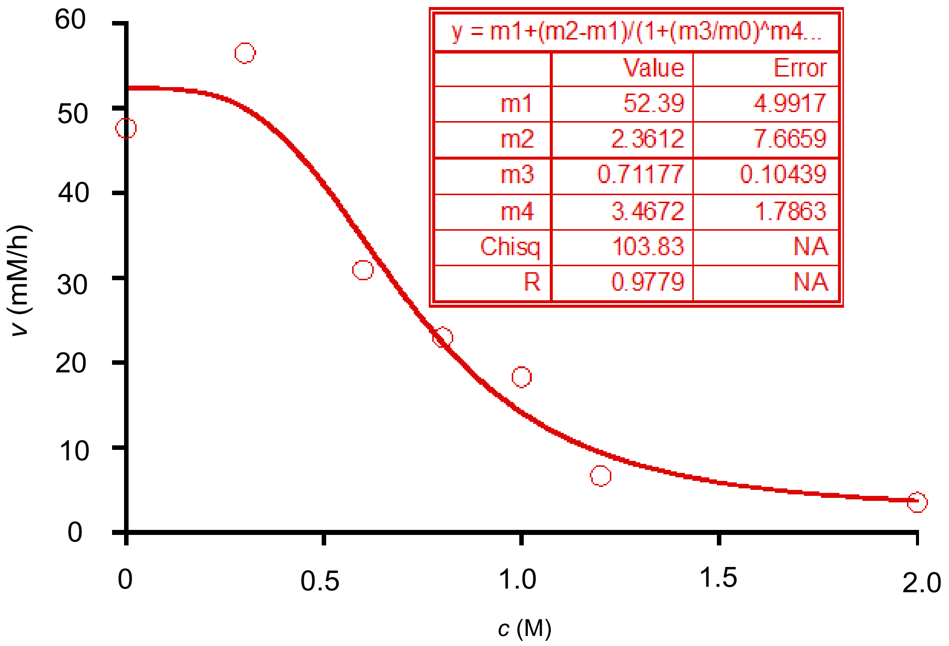

Figure S17. Hill equation fitting for plot of velocities of the reaction catalyzed by **5** against the concentration of $TBANO_3$.

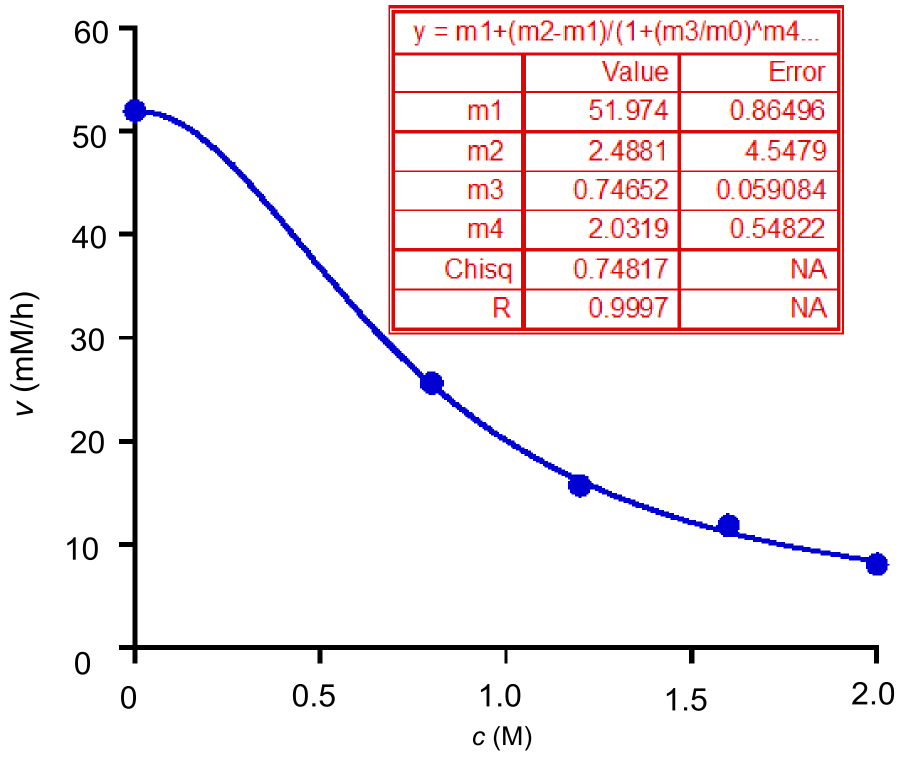

Figure S18. Hill equation fitting for plot of vilocities of the reaction catalyzed by **13** against the concentration of $TBANO_3$.

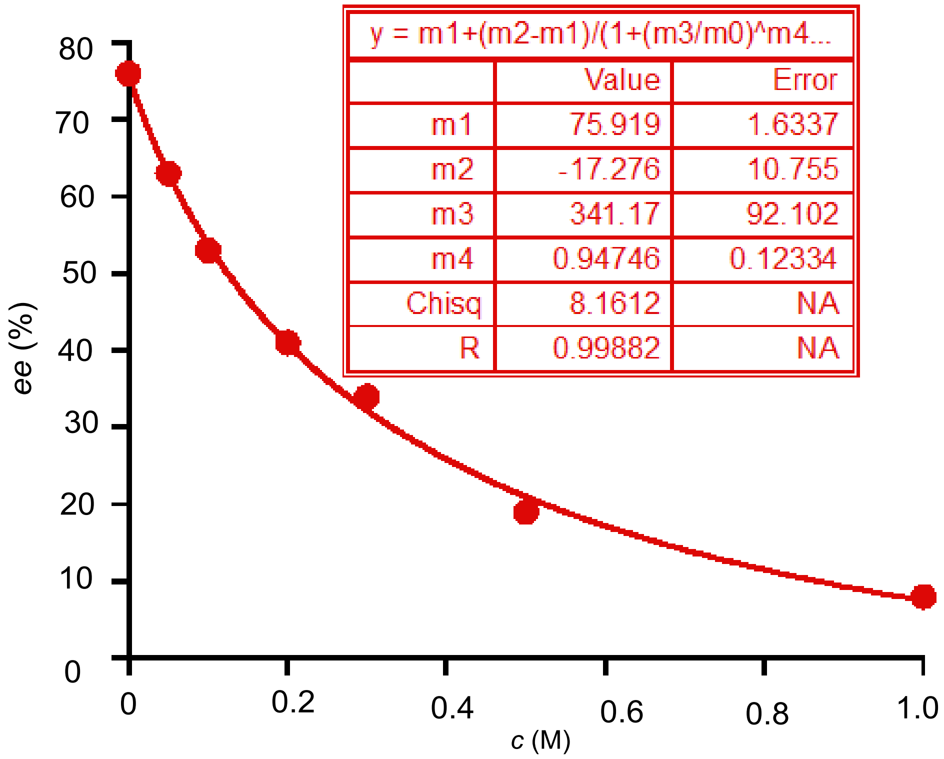

Figure S19. Dependence of the *ee* with S112W + **16** on the concentration of $NaNO_3$.

Table S1 Results of the kinetics measurements.

entry[a]	catalyst[b]	*η* (%)[c]	*dr*[d]	*ee* (%)[e]	v/v_0[f]	ΔE_a (kJ mol^{-1})[g]
1	**11 + 12**	95	7:1	54	-	-
2	**5**	91	9:1	86	12	6.1
3	**14**	90	4:1	-60	-	-
4	**13**	89	>20:1	-94	10	5.7
5	**5**	91	9:1	86	-	-
6	**5 + PF_6^-**	90	>20:1	90	0.81	0.5
7	**5 + BF_4^-**	89	>20:1	85	0.54	1.5
8	**5 + Br^-**	94	>20:1	85	0.28	3.1
9	**5 + NO_3^-**	89	>20:1	78	0.13	4.9
10	**11 + 12**	95	7:1	54	-	-
11	**11 + 12+ PF_6^-**	94	10:1	30	0.98	0.1
12	**11 + 12+ NO_3^-**	92	8:1	52	0.89	0.3

[a]Catalysts, see Figure 2. [b]400 mM **1**, 800 mM **2**, 2.5-10 mol% catalyst, 20 ℃, 48 h, [c]Yield was determined based on crude ^{1}H NMR spectroscopy with dibromomethane as internal standard. [d]Diastereomeric ratio based on crude ^{1}H NMR spectroscopy; [e]Enantiomeric excess. Positive values refer to **3**, negative values to **3e**, Figure 1. [f]Determined from the linear fitting, $v_0 = v_{\mathbf{11+12}}$ for catalyst **5**, $v_0 = v_{\mathbf{14}}$ for catalyst **13**; for the inhibition reactions, v_0 refers to the velocity of the reaction without addition of TBA salts. [g] transition-state stabilization, $\Delta E_a = -RT \ln(v_{ini}/v_{ini}^0)$

Table S2 Streptavidin library screening.

entry[a]	protein[b]	η (%)[c]	*ee* (%)[d]
1	WT	53%	45%
2	S112Y	47%	53%
3	S112W	50%	76%
4	S112F	47%	53%
5	S112H	50%	32%
6	S112E	51%	24%
7	K121R	47%	20%
8	K121A	39%	0%
9	K121H	36%	10%
10	K121R	35%	0%
11	L124Y	43%	10%
12	L124F	60%	39%
13	S112A-K121A	42%	0%
14	S112Y-K121E	37%	10%
15	S112Y-K121R	47%	53%

[a]10 μM **1**, 25 μM **2**, 0.1 μM biotinylated catalyst **16** mixed with the protein (200 μL, 1 mM, Bis-Tris pH 6.5, 0.2 μmol) and stirred at 20 ℃. [b]Streptavidin, WT = wild type. [c]Conversion was determined based on crude ^{1}H NMR spectroscopy with dibromomethane as internal standard. [d]Enantiomeric excess.

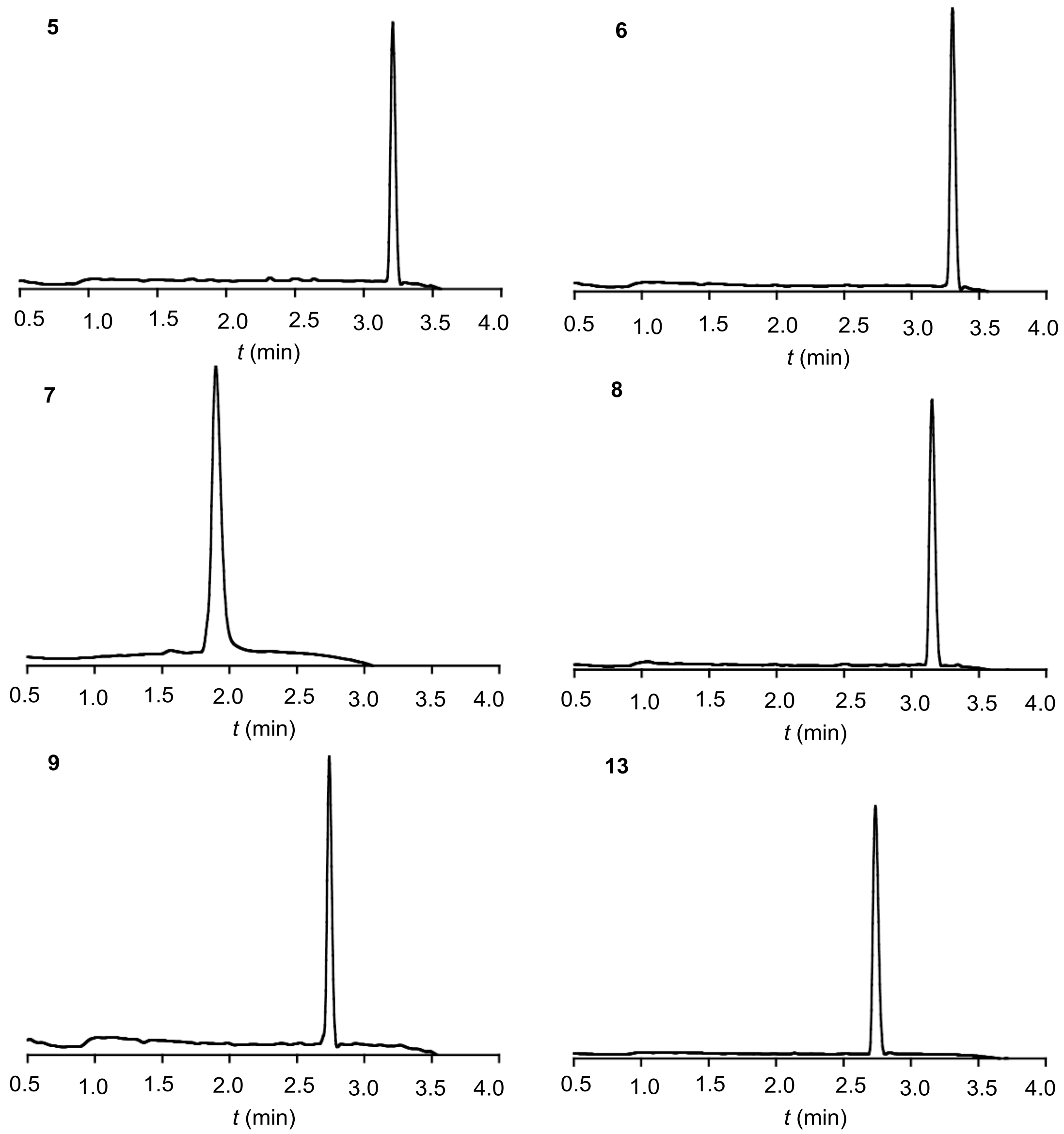

Figure S20. Reverse phase HPLC analyses of catalysts obtained using a Thermo C18 (5 cm x 2.1 mm, 1.9 µm particles) Hypersil gold column with a linear elution gradient from 5% to 90% CH_3CN/H_2O with 0.01% TFA in 4.0 minutes at a flow rate of 0.75 mL/min, detected by absorbance at 190 nm to 800 nm.

5. References

S1 Y. Cotelle, S. Benz, A.-J. Avestro, T. R. Ward, N. Sakai and S. Matile, *Angew. Chem. Int. Ed.*, 2016, **55**, 4275–4279.

S2 L. Liu and S. Matile, *Supramol. Chem.*, 2017, DOI: 10.1080/10610278.2016. 1258118.

S3 Y. Cotelle, V. Lebrun, N. Sakai, T. R. Ward and S. Matile, *ACS Cent. Sci*., 2016, **2**, 388–393.

S4 A. A. Berezin, A. Sciutto, N. Demitri and D. Bonifazi, *Org. Lett*., 2015, **17**, 1870–1873.

S5 J. Huang, G. Chen, X. Fu, C. Li, C. Wu and Q. Miao, *Catal. Sci. Technol*., 2012, **2**, 547–553.

S6 S. Lee, Y. Hua and A. H. Flood, *J. Org. Chem.*, 2014, **79**, 8383–8396.

S7 F. N. Miros, Y. Zhao, G. Sargsyan, M. Pupier, C. Besnard, C. Beuchat, J. Mareda, N. Sakai and S. Matile, *Chem. Eur. J.,* 2016, **22**, 2648–2657.

S8 M. Akamatsu and S. Matile, *Synlett*, 2016, **27**, 1041–1046.

S9 C. Cassani, R. Martin-Rapun, E. Arceo, F. Bravo and P. Melchiorre, *Nat. Protoc.*, 2013, **8**, 325–344.

S10 M. Rueping, A. Kuenkel and R. Frohlich, *Chem. Eur. J*., 2010, **16**, 4173–4176.

S11 D. Ding, C.-G. Zhao, Q. Guo and H. Arman, *Tetrahedron*, 2010, **66**, 4423–4427.

S12 M. Skander, N. Humbert, J. Collot, J. Gradinaru, G. Klein, A. Loosli, J. Sauser, A. Zocchi, F. Gilardoni and T. R. Ward, *J. Am. Chem. Soc.*, 2004, **126**, 14411–14418.

S13 M. Creus, A. Pordea, T. Rossel, A. Sardo, C. Letondor, A. Ivanova, I. LeTrong, R. E. Stenkamp and T. R. Ward, *Angew. Chem. Int. Ed.*, 2008, **47**, 1400–1404.

6. NMR Spectra

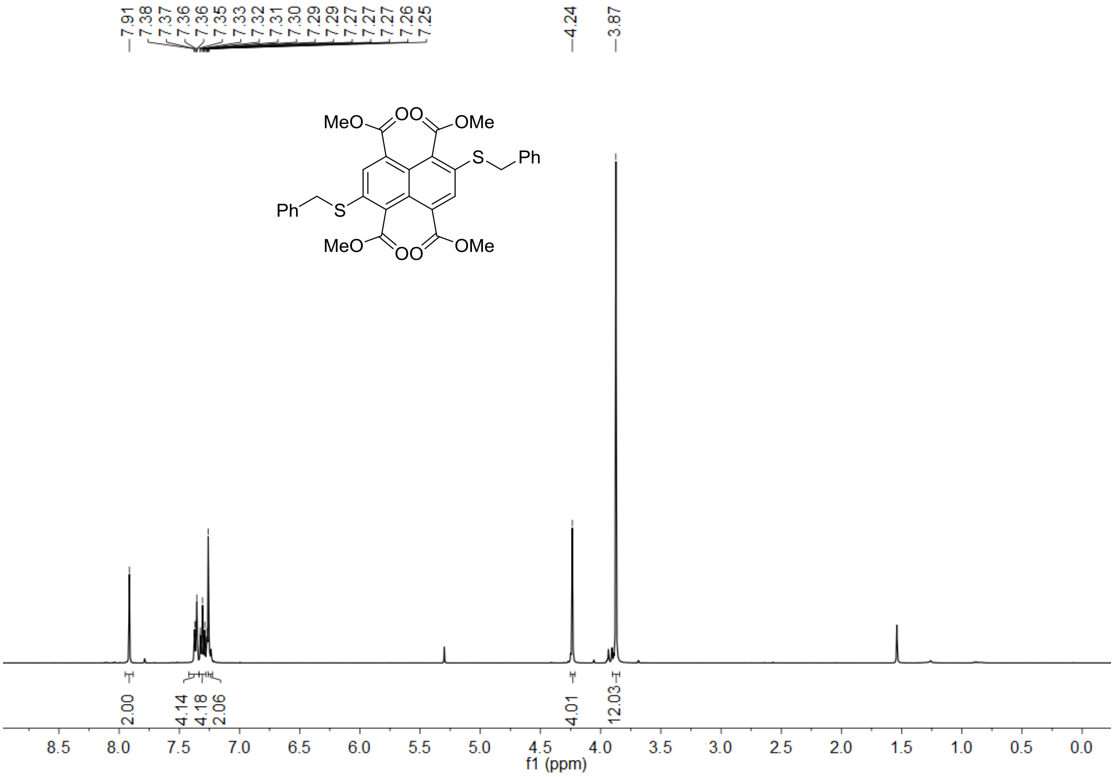

Figure S21. ^{1}H NMR spectrum of **18** in $CDCl_3$.

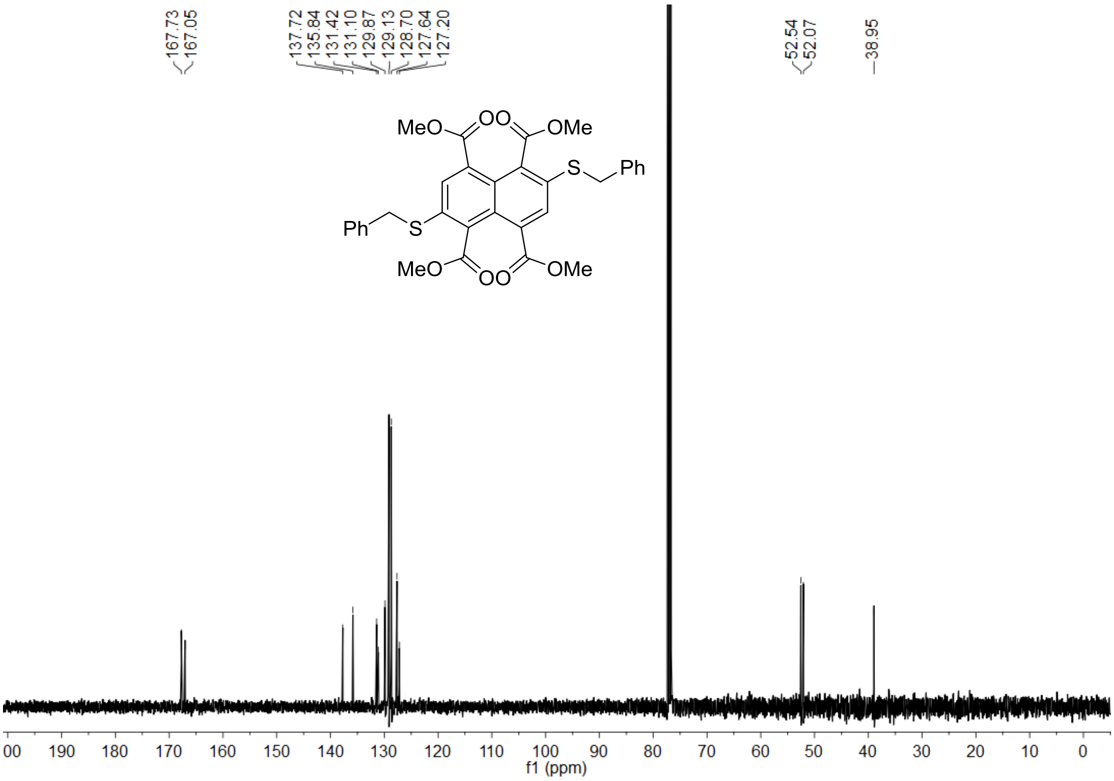

Figure S22. ^{13}C NMR spectrum of **18** in $CDCl_3$.

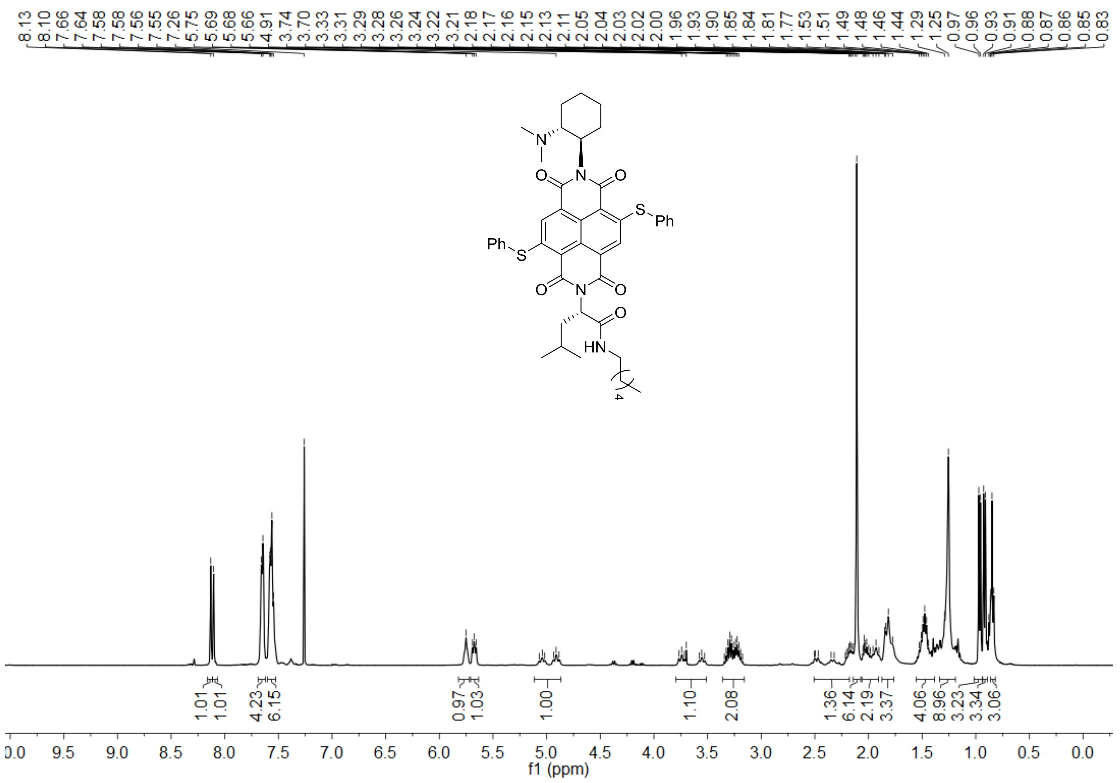

Figure S23. ^{1}H NMR spectrum of **5** in $CDCl_3$.

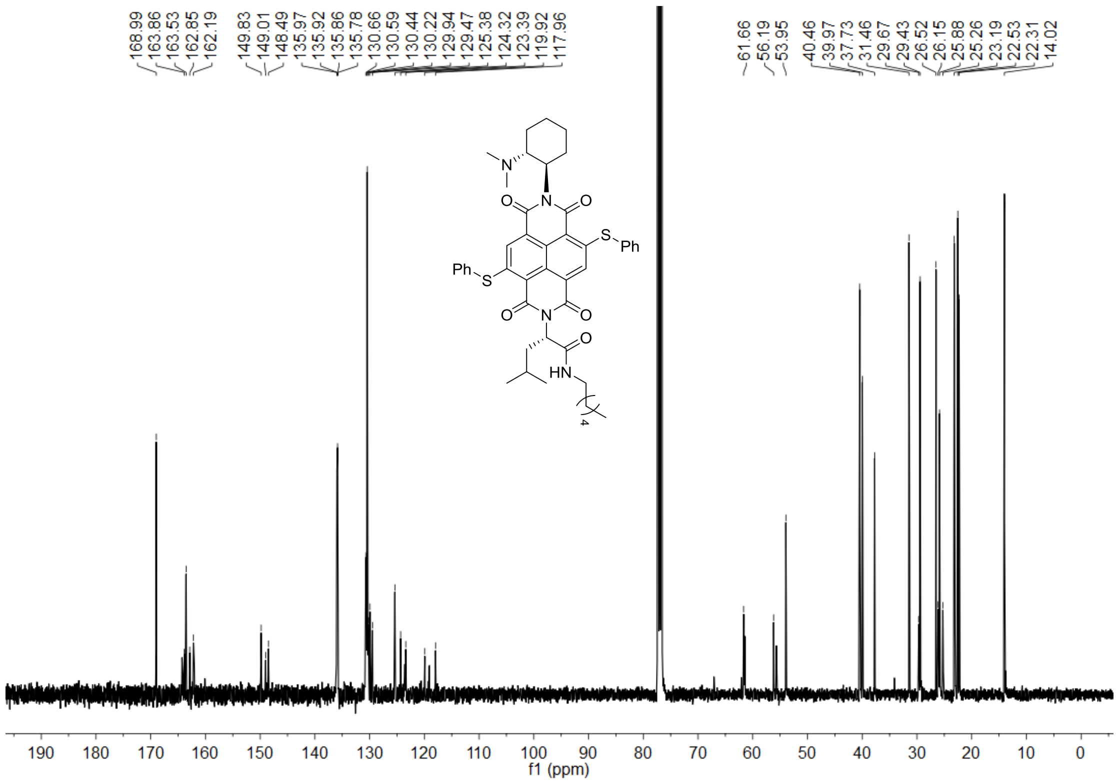

Figure S24. ^{13}C NMR spectrum of **5** in $CDCl_3$.

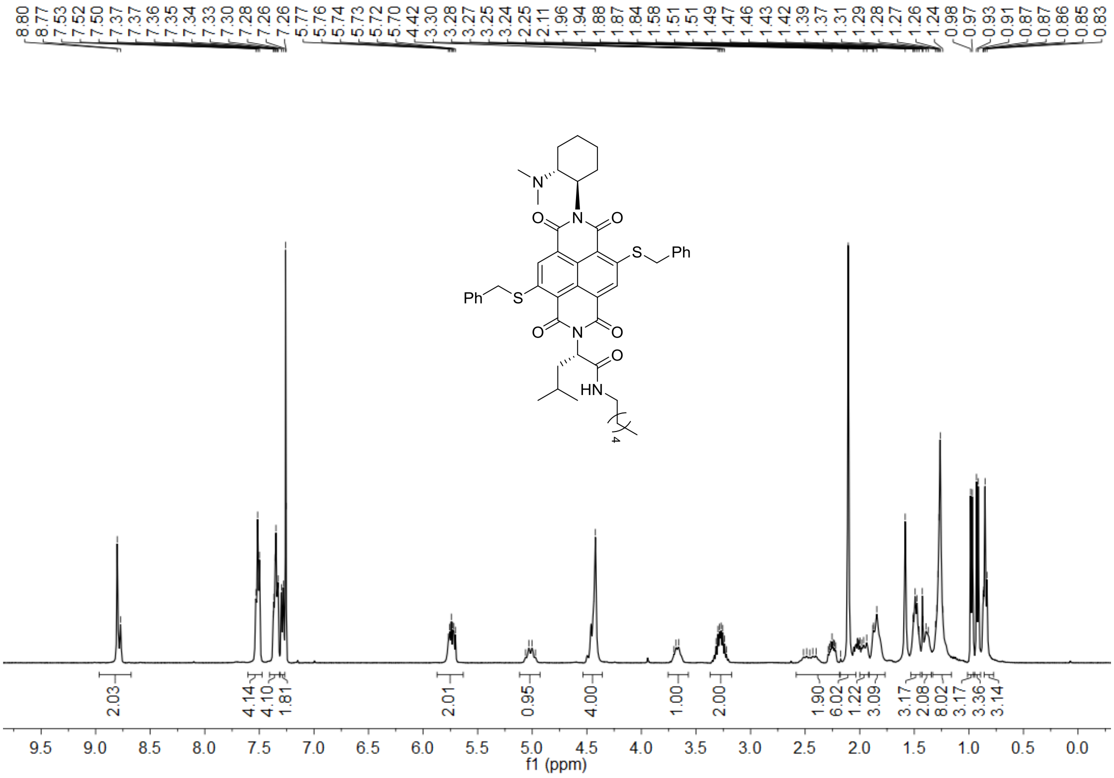

Figure S25. ^{1}H NMR spectrum of **6** in $CDCl_3$.

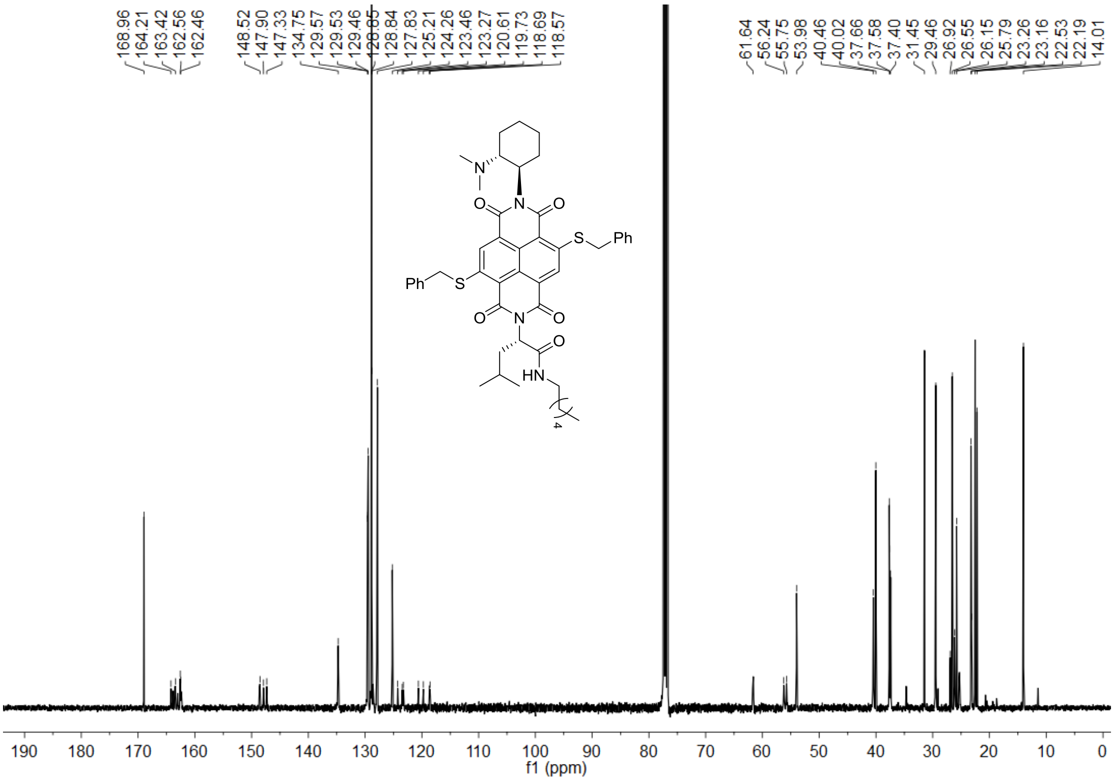

Figure S26. ^{13}C NMR spectrum of **6** in $CDCl_3$.

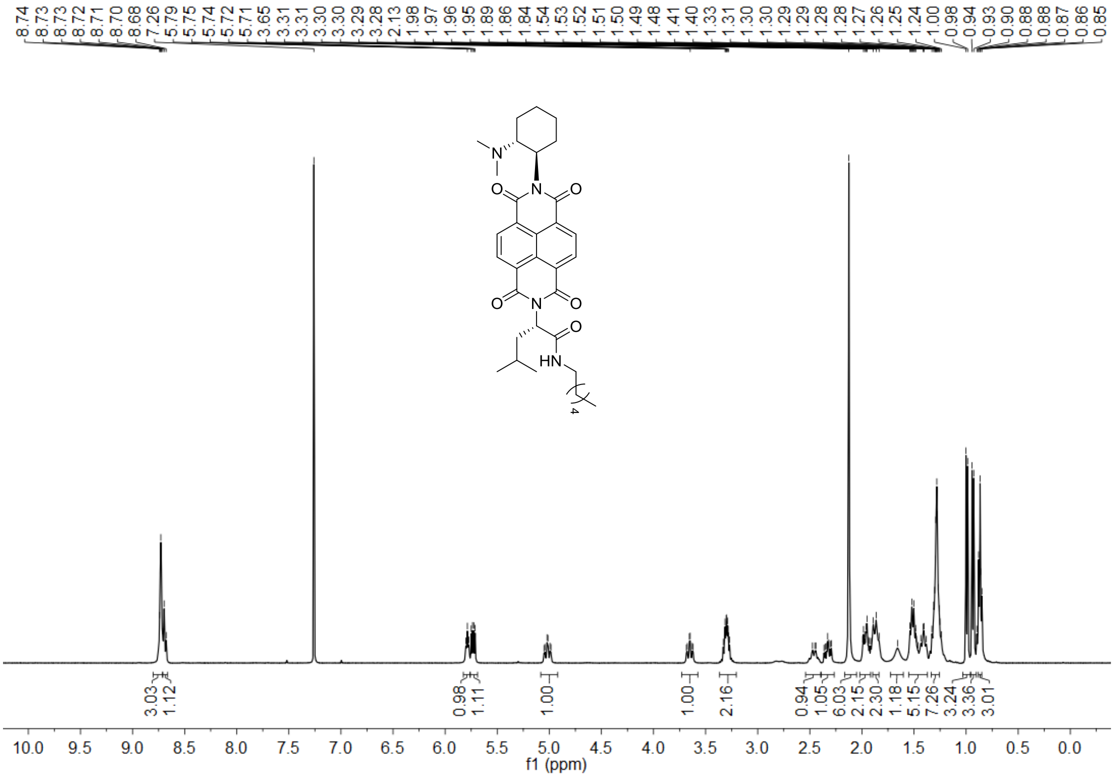

Figure S27. ^{1}H NMR spectrum of **7** in $CDCl_3$.

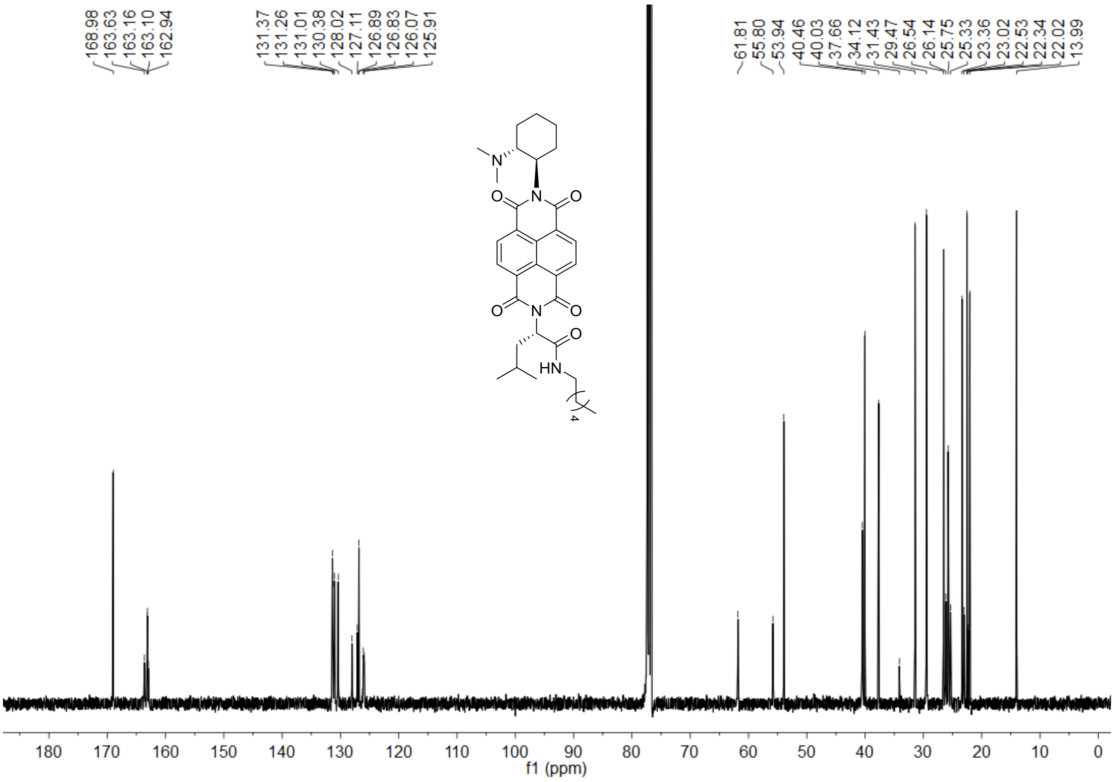

Figure S28. ^{13}C NMR spectrum of **7** in $CDCl_3$.

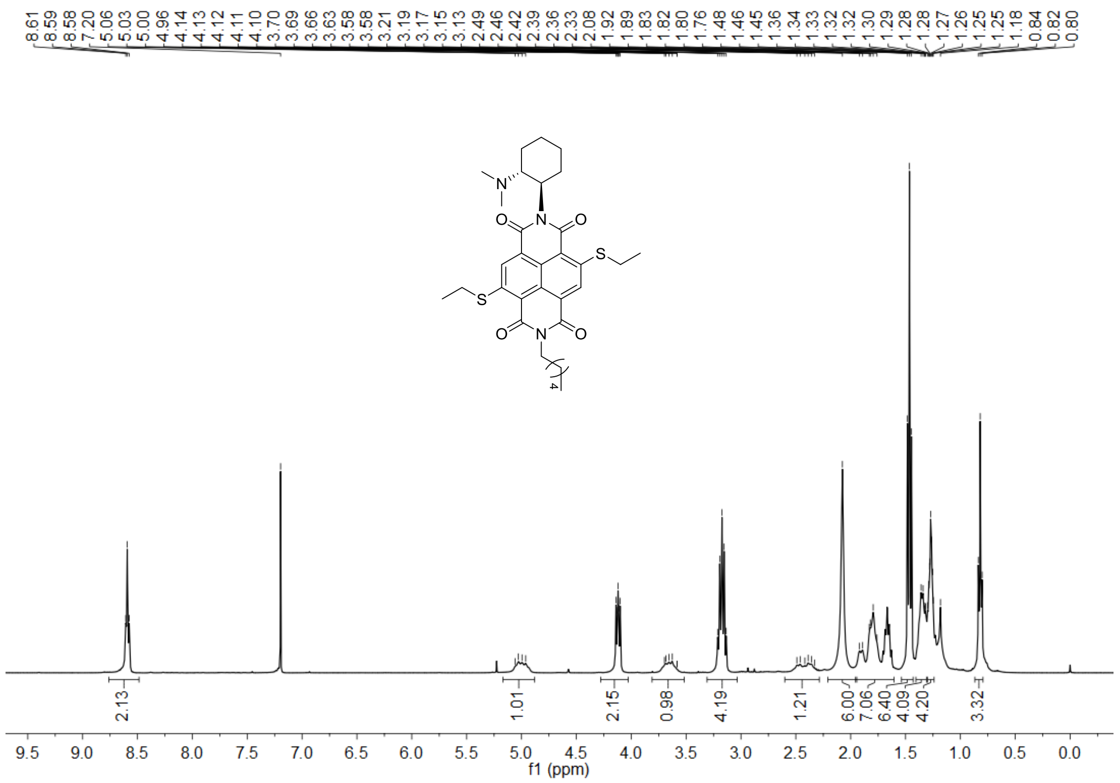

Figure S29. ^{1}H NMR spectrum of **8** in $CDCl_3$.

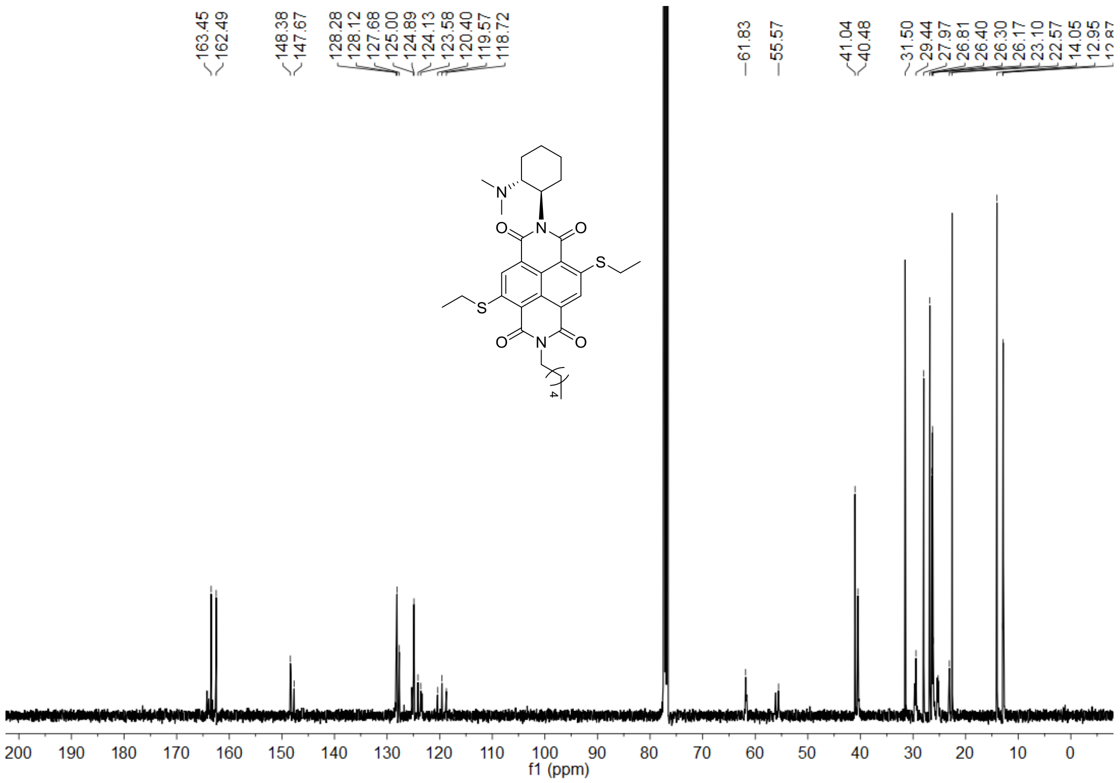

Figure S30. ^{13}C NMR spectrum of **8** in $CDCl_3$.

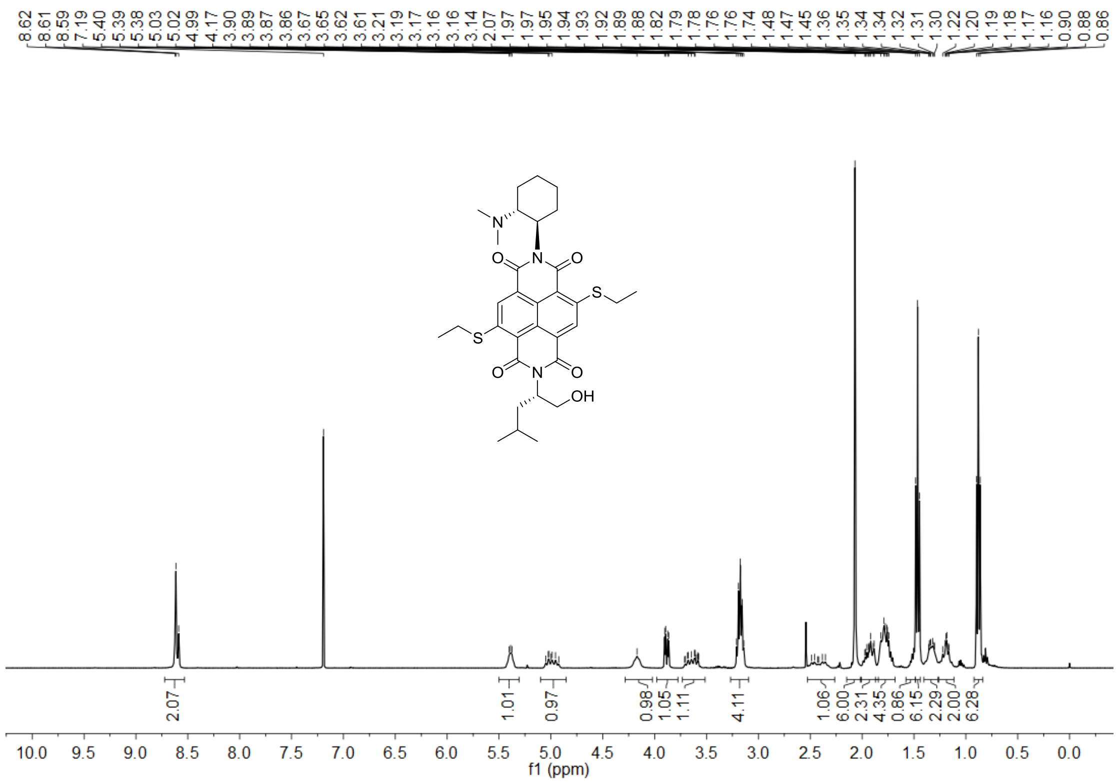

Figure S31. ^{1}H NMR spectrum of **9** in $CDCl_3$.

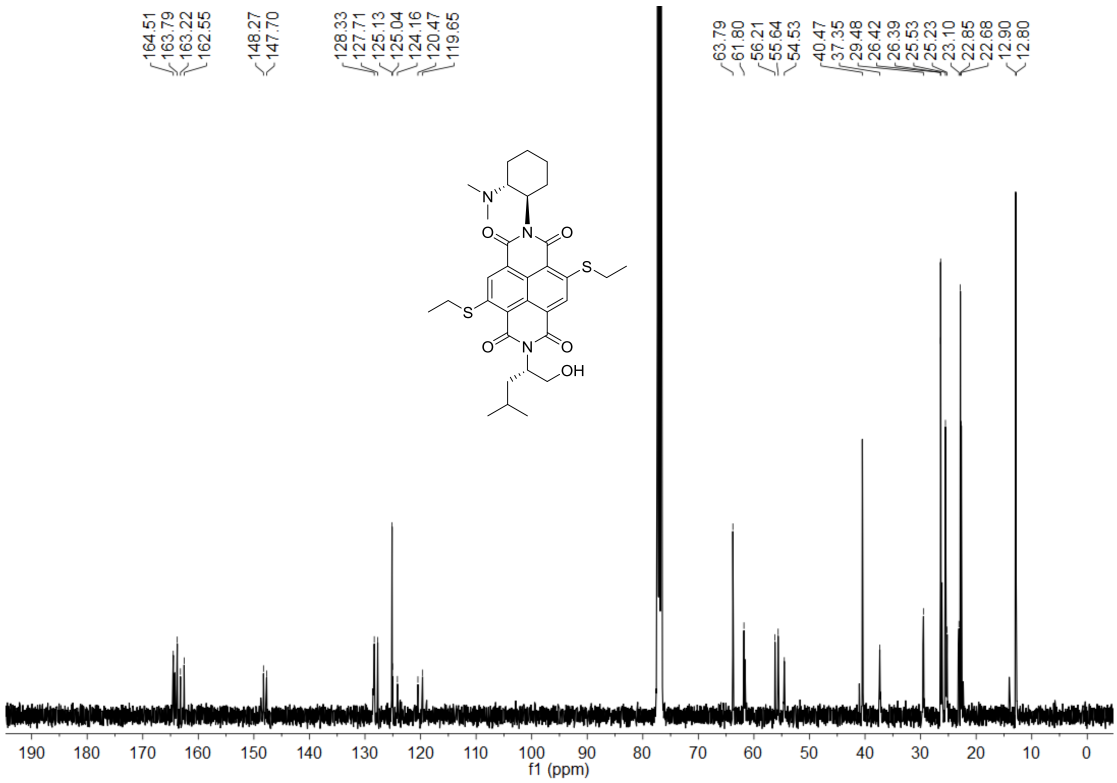

Figure S32. ^{13}C NMR spectrum of **9** in $CDCl_3$.

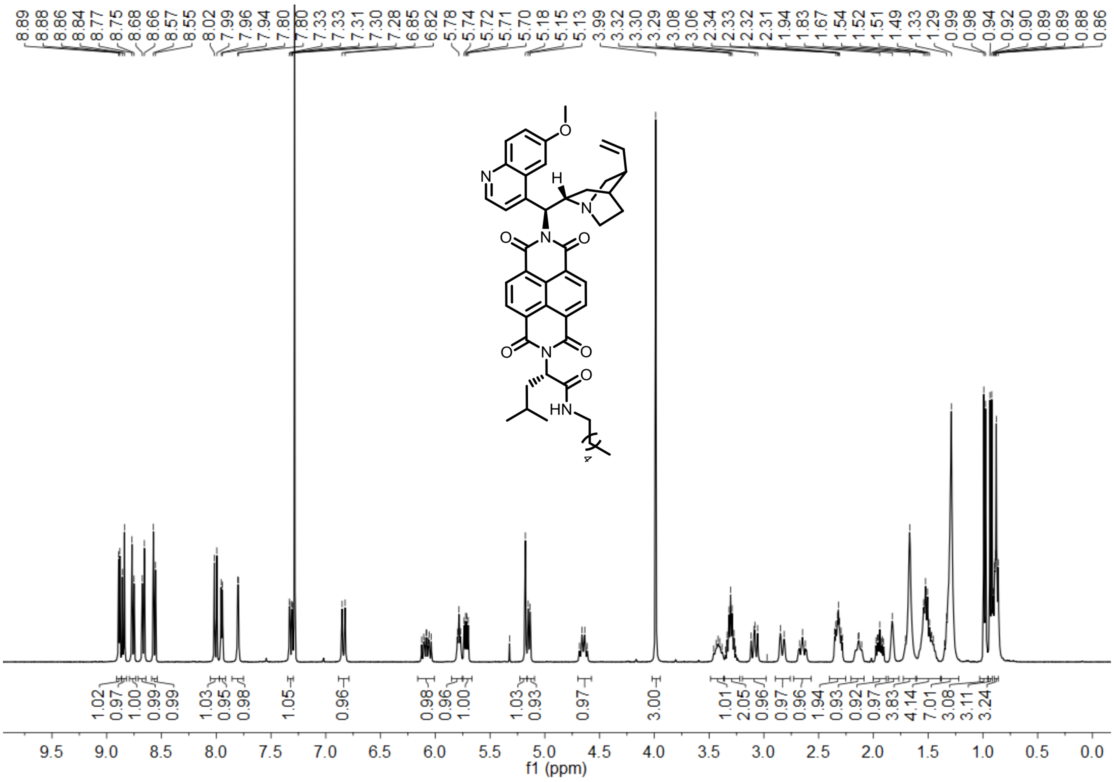

Figure S33. ^{1}H NMR spectrum of **13** in $CDCl_3$.

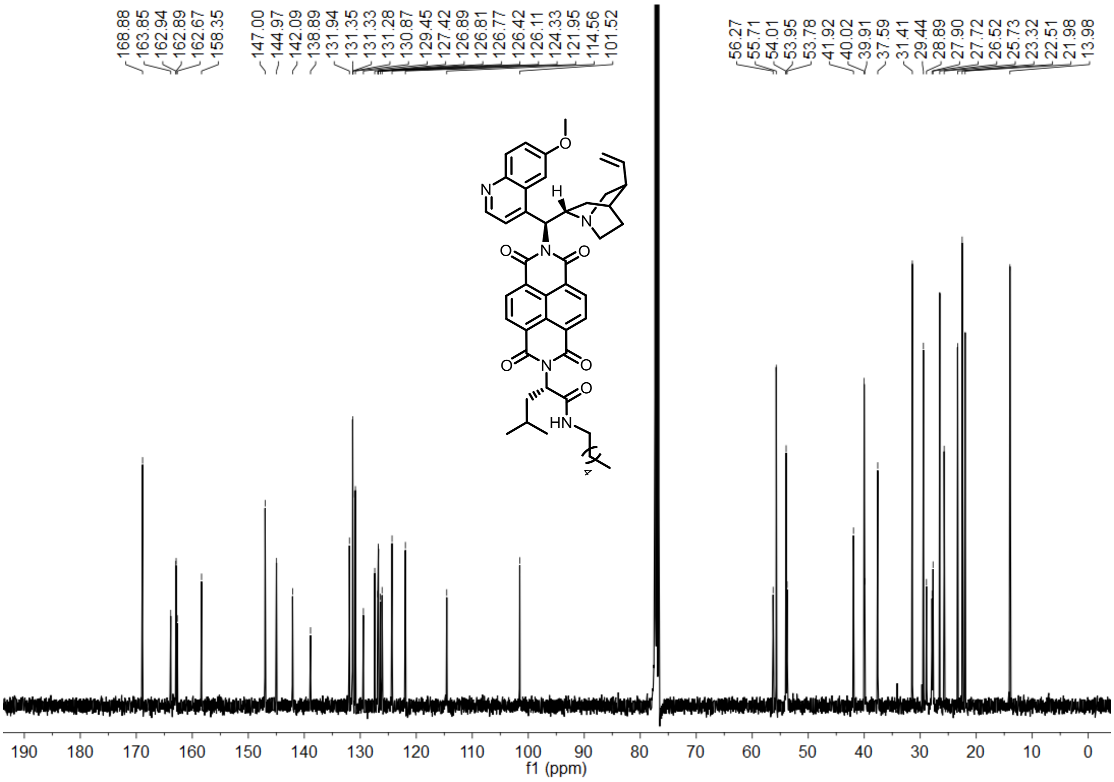

Figure S34. ^{13}C NMR spectrum of **13** in $CDCl_3$.

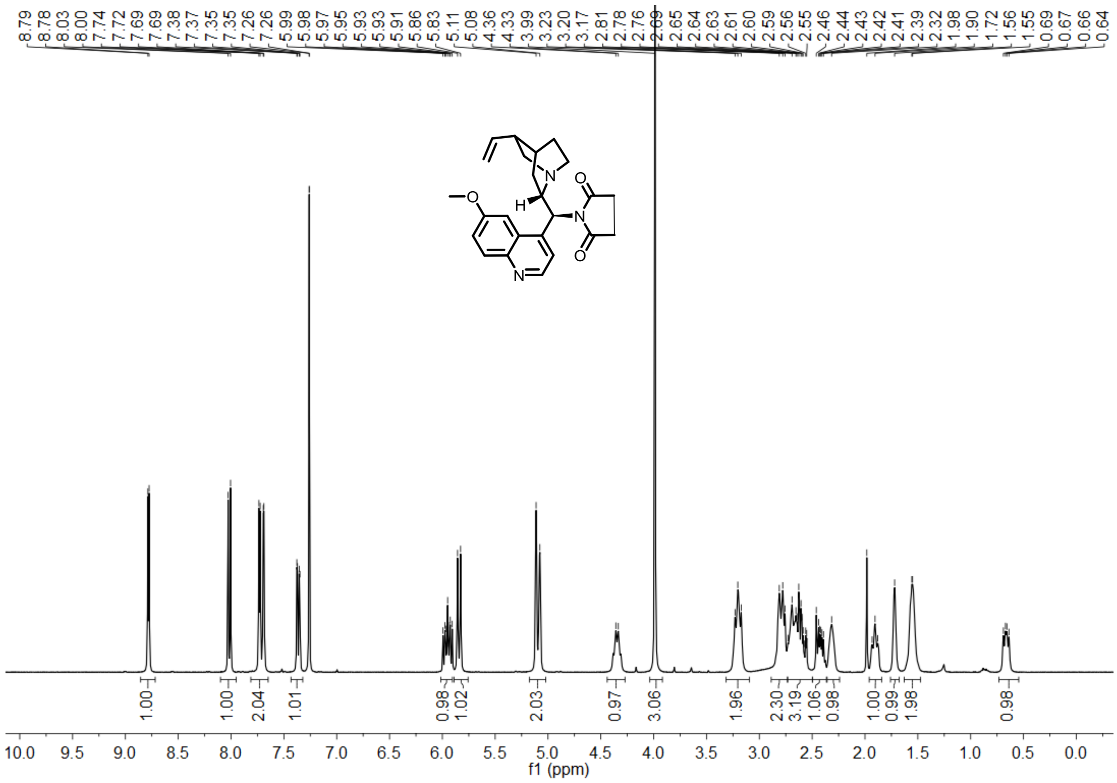

Figure S35. ^{1}H NMR spectrum of **14** in $CDCl_3$.

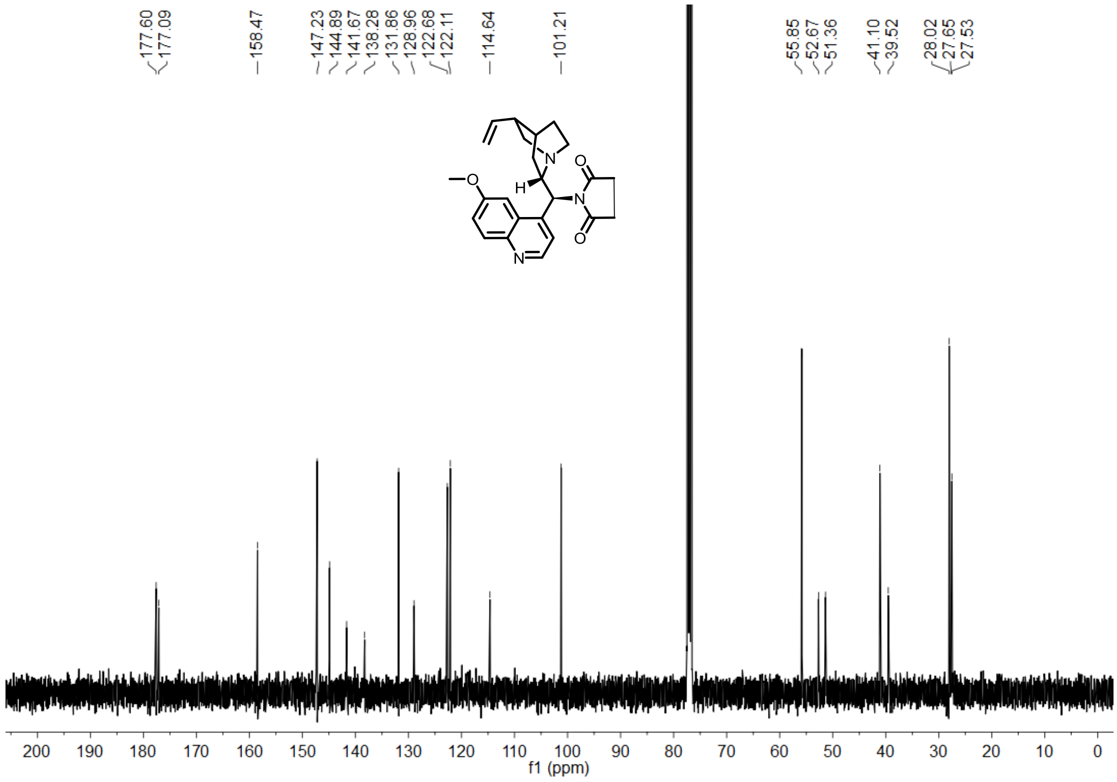

Figure S36. ^{13}C NMR spectrum of **14** in $CDCl_3$.
